# Observation of thermally stable polar metallic state in perovskite nitride CeWN_3_

**DOI:** 10.1126/sciadv.aeh6191

**Published:** 2026-09-25

**Authors:** Xubin Ye, Shaoxuan Zheng, Zhaoliang Chen, Zunyi Deng, Zhiyu Liao, Xianggang Qiu, Xiao Wang, Zhiwei Hu, Chang-Yang Kuo, Chien-Te Chen, Chih-Wen Pao, Cheng Dong, Zhao Pan, Jiawang Hong, Lin Gu, Zhen Chen, Youwen Long

**Affiliations:** ^1^ Beijing National Laboratory for Condensed Matter Physics, Institute of Physics, Chinese Academy of Sciences, Beijing 100190, China.; ^2^ School of Materials Science and Engineering, Tsinghua University, Beijing 100084, China.; ^3^ School of Aerospace Engineering, Beijing Institute of Technology, Beijing 100081, China.; ^4^ School of Physical Sciences, University of Chinese Academy of Sciences, Beijing 100049, China.; ^5^ Max-Planck Institute for Chemical Physics of Solids, Nöthnitzer Straße 40, Dresden 01187, Germany.; ^6^ National Synchrotron Radiation Research Center, Hsinchu 30076, Taiwan.; ^7^ Department of Electrophysics and Center for Emergent Functional Matter Science, National Yang Ming Chiao Tung University, Hsinchu 30093, Taiwan.

## Abstract

Polar metals offer substantial potential for exotic quantum phenomena and multifunctional applications. However, the existence of a polar metal is fundamentally challenging owing to the screening of dipole-dipole interactions by conducting electrons. Specifically, a high-temperature–stabilized polar metal remains to be discovered. In this study, we report a thermally stable polar-metal state in perovskite cerium tungsten nitride. Synchrotron x-ray diffraction and electron microscopy reveal a polar *Pna*2_1_ structure, while second-harmonic generation demonstrates that the polar response persists up to 850 kelvins in argon. Electrical resistivity and optical conductivity measurements demonstrate metallic transport behavior, consistent with the itinerant electronic contribution derived from specific heat analysis. First-principles calculations reveal that the polar distortion and metallic conductivity originate from tungsten-centered nitrogen octahedra, where the off-center displacements of hexavalent tungsten ions govern polarity and hybridization between tungsten 5d and nitrogen 2p states contributes to itinerant carriers. This study establishes a record-high-temperature polar metal, opening a promising avenue for exploring robust polar and metallic materials in perovskite nitrides.

## INTRODUCTION

Polar metals combine long-range polar distortion with metallic conduction within single-phase materials (*1*, *2*). By integrating broken inversion symmetry with itinerant carriers, these materials provide numerous opportunities for a wide variety of inversion-symmetry–forbidden quantum phenomena (*3*–*6*) such as Rashba spin splitting for spintronic control (*7*), Berry-curvature–driven nonlinear Hall responses (*8*, *9*), and even parity-mixed or ferroelectric-like superconductivity (*10*). However, reconciling these two attributes is extremely difficult because classical ferroelectrics sustain polarity through cooperative and symmetry-breaking displacements that generate permanent dipoles, whereas metals favor high-symmetry lattices whose itinerant electrons screen the internal electric fields for long-range dipole interactions. Consequently, polarity in metallic systems does not necessarily imply switchable ferroelectric polarization. Beyond electrostatic screening, a deeper conflict is rooted in the electronic bonding framework (*6*, *11*–*15*). Metallic bonding, which is spatially uniform and isotropic, is inherently incompatible with the directional and anisotropic distortions required for polarity. This essential contradiction constitutes a core bottleneck for realizing a robust polar metal state, unless mitigated by orbital-selective hybridization and/or the coexistence of distinct bonding motifs. For example, in the prototypical polar metal LiOsO_3_ (*16*–*19*), a continuous noncentrosymmetric distortion emerges below 140 K from local Li^+^ off-centering, whereas itinerant electrons are hosted in the OsO_6_ sublattice. In layered WTe_2_, the polarity originates from low-symmetry stacking combined with strong spin-orbit coupling (*1*), yielding broken inversion symmetry and topologically metallic nontrivial bands. More recently, studies on Ruddlesden-Popper cobaltates have demonstrated that geometric asymmetry and ordered oxygen vacancies can induce polar distortions in layered structures (*20*, *21*) and strong 3d-2p hybridization stabilizes the metallicity in a polar lattice. These systems exemplify the coexistence of local bonding asymmetry and itinerant electronic states via mixed bonding architectures. Nevertheless, in these cases, the polar metal states are often limited to marginal instabilities, low transition temperatures, or delicate stoichiometric tuning (*22*). However, the realization of thermodynamically stable polar metals at high temperatures remains challenging.

Compared with the widely examined ABO_3_ perovskite oxides, ABN_3_ perovskite nitrides remain largely underexplored in experiments (*23*–*26*) because of synthetic challenges such as the requirement for high nitrogen chemical potential to stabilize high-valent nitrides and their sensitivity to air and moisture. Now, a few ABN_3_ thin films, such as AWN_3_ (A = La, Ce, and Gd) and CeBN_3_ (B = Mo and Ta), have been reported (*23*, *27*–*29*). These films often suffer from nonstoichiometry and ambiguous crystal symmetries. For example, CeWN_3_ thin films produced via high-throughput reactive sputtering readily form mixed perovskite- and fluorite-family phases with nitrogen deficiency and an unresolved crystal structure (*28*). Recent advances in high-pressure synthesis have opened a pathway for obtaining bulk ABN_3_ perovskites with improved crystallinities and broad electronic diversity (*30*–*39*), as demonstrated in ATaN_3_ (A = Th and Ce) and LaBN_3_ (B = W and Re) (*31*, *32*, *40*–*43*). The presence of polar structure in nitrogen-deficient LaWN_3−δ_ (δ ≈ 0.5) provides opportunities to realize a possible polar metallic state in perovskite nitrides (*23*, *31*). Leveraging the unique superiority of high-pressure synthesis techniques for perovskite nitrides, chemically stoichiometric high-quality bulk CeWN_3_ with a polar *Pna*2_1_ structure was synthesized for the first time at 5 GPa and 1773 K. Comprehensive structural and physical analyses revealed its robust polarity and intrinsic metallicity across a wide temperature range of up to 850 K in argon, representing a record-high-temperature polar metal.

## RESULTS AND DISCUSSION

### High-pressure synthesis and crystal structure

**Fig. 1.**
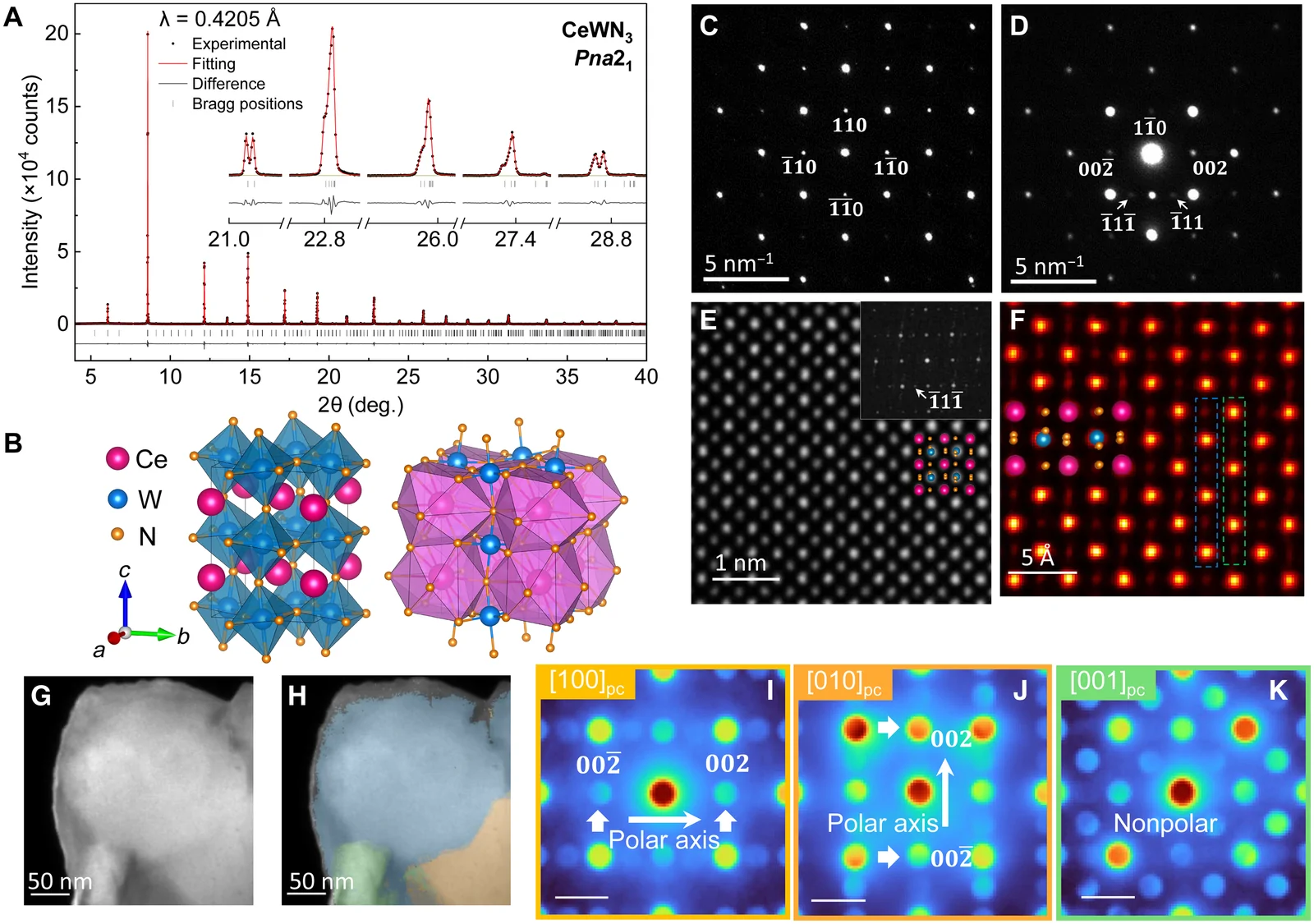
Room-temperature crystal structural characterizations of CeWN_3_. (**A**) Synchrotron x-ray diffraction (SXRD) pattern and Rietveld refinement (*Pna*2_1_ model). Inset: Enlarged view of selected 2θ regions showing excellent agreement. (**B**) Polyhedral view of the CeWN_3_ framework. Selected-area electron diffraction (SAED) patterns along (**C**) [001] (pseudocubic [001]_pc_), and (**D**) [110] (pseudocubic [100]_pc_) zone axes where ($\bar{1}1\bar{1}$) and ($\bar{1}11$) reflections are shown. (**E**) High-angle annular dark-field scanning transmission electron microscopy (HAADF-STEM) image along [110], showing strong atomic number contrast that highlights Ce and W atomic columns. (**F**) Projected multislice electron ptychography (MEP) phase image along [110], resolving N positions. Green and blue dashed rectangles highlight two distinct arrangements of neighboring N rows along the *c* axis. (**G**) Virtual ADF image generated from 4D-STEM dataset. (**H**) Domains of two zone axes derived from STEM–nanobeam electron diffraction (NBED) data. The blue and orange regions correspond to the polar [110] zone axis (pseudocubic [100]_pc_) but represent domains with orthogonal polarization directions: The polar [001] axis is aligned horizontally in the blue regions and vertically in the orange regions. The green regions correspond to the nonpolar [001] zone axis (pseudocubic [001]_pc_). (**I** to **K**) Representative diffraction patterns extracted from the blue, orange, and green regions in (H), respectively.

Figure 1A shows the powder synchrotron x-ray diffraction (SXRD) pattern of the high-pressure–synthesized CeWN_3_ acquired at room temperature (RT). The presence of superstructure reflections and systematic splitting of the pseudocubic peaks (Fig. 1A, inset) illustrates unambiguous structural distortions. All the diffraction peaks can be indexed to a primitive orthorhombic lattice, and no discernible impurities are observed. Further Rietveld refinements against structural models, as previously reported for its analog LaWN_3−δ_ bulk (*23*, *28*, *31*, *42*), reveal that the polar *Pna*2_1_ (no. 33) space group provides a good fit to the SXRD data (*R*_wp_ = 6.80%, *R*_p_ = 4.78%, and χ^2^ = 1.88), yielding lattice constants of *a* = 5.61525(1) Å, *b* = 5.62785(1) Å, and *c* = 7.94437(2) Å. Detailed structural parameters, bond lengths, and bond angles are summarized in tables S1 and S2. Figure 1B schematically illustrates the crystal structure of CeWN_3_, in which corner-sharing WN_6_ octahedra form the fundamental framework and Ce cations occupy the interstitial A sites.

To further confirm the polar structure, additional structural characterization was performed using electron microscopy. The selected-area electron diffraction (SAED) patterns acquired along the pseudocubic [001]_pc_ and [100]_pc_ zone axes (Fig. 1, C and D) exhibit the characteristic extinction conditions [*h*00]/[0 *k*0]/[00 *l*] (*h*, *k*, *l* = 2*n* + 1). Additionally, the presence of ($\bar{1}1\bar{1}$) and ($\bar{1}11$) reflections in the pseudocubic [001]_pc_ zone axis highlights the unique periodicity along the *c* axis. These symmetrical features are in good agreement with the simulated diffraction patterns based on the *Pna*2_1_ model (fig. S1). A high-angle annular dark-field scanning transmission electron microscopy (HAADF-STEM) image along the [100]_pc_ direction (Fig. 1E) resolves the Ce and W atomic columns, which is consistent with the refined structure. The corresponding multislice electron ptychography (MEP) phase image (Fig. 1F) further reveals well-defined, elongated nitrogen atomic positions, indicating alternating stacking sequences of neighboring N rows along the *c* axis (highlighted by dashed rectangles), consistent with the distorted polar perovskite structure.

### Polarity characterizations

Experimentally, distinguishing the [001] zone axis of CeWN_3_ (corresponding to the pseudocubic [001]_pc_, which appears to be nonpolar) from the [110] zone axis (corresponding to the pseudocubic [100]_pc_, which contains polarity) is challenging in real space because of their similar atomic arrangements. However, these orientations can be unambiguously distinguished by their unique diffraction patterns in the reciprocal space. To this end, we performed a four-dimensional (4D) STEM experiment using an electron microscope pixel array detector (EMPAD) to record 2D nanobeam electron diffraction (NBED) patterns at each probe position during 2D scanning of probe positions, as illustrated in fig. S2 (for details, refer to Materials and Methods), resulting in 4D datasets (*44*). Figure 1G shows a virtual ADF image reconstructed by integrating the diffraction intensity within a certain annular range from the 4D dataset, illustrating the general morphology of the sample flake. Analysis of the dataset revealed the coexistence of regions oriented along the [001] (nonpolar projection) and [110] (polar projection) axes, which are color coded in Fig. 1H, and their corresponding representative diffraction patterns are displayed in Fig. 1 (I to K) and fig. S3. Figure 1 (I and J) shows the diffraction patterns for the [110] orientation with perpendicular polarization directions, and Fig. 1K shows the pattern for the [001] orientation, which is parallel to the polarization axis. Furthermore, owing to dynamic diffraction effects, polar structural distortion causes a breakdown of Friedel’s law, resulting in an intensity discrepancy between symmetric diffraction disks along the polar axis (*45*, *46*). This effect is evident in the on-axis NBED pattern shown in Fig. 1I and fig. S4, where pronounced intensity asymmetry exists between (002) and ($00\bar{2}$) reflections, closely matching simulations based on the polar *Pna*2_1_ model. This is an unambiguous signature of inversion symmetry breaking owing to Friedel’s law breaking, further affirming the polar character of CeWN_3_. Additionally, scanning electron microscopy (fig. S5A) shows well-formed crystalline grains with typical sizes of ∼10 μm. Elemental mapping and line-scan energy-dispersive x-ray spectroscopy (EDS) profiles (fig. S5, C and D) confirm the homogeneous spatial distribution of Ce, W, and N, with an overall elemental ratio matching the nominal 1:1:3 stoichiometry within the instrumental precision. The O mapping (fig. S5B) shows only a weak background-level signal without obvious oxygen enrichment correlated with the crystal. Moreover, x-ray absorption spectroscopy (XAS; Supplementary Text and fig. S6) revealed a Ce^3+^/W^6+^ valence configuration (*47*, *48*), further confirming the chemical stoichiometry of the high-pressure–synthesized CeWN_3_.

**Fig. 2.**
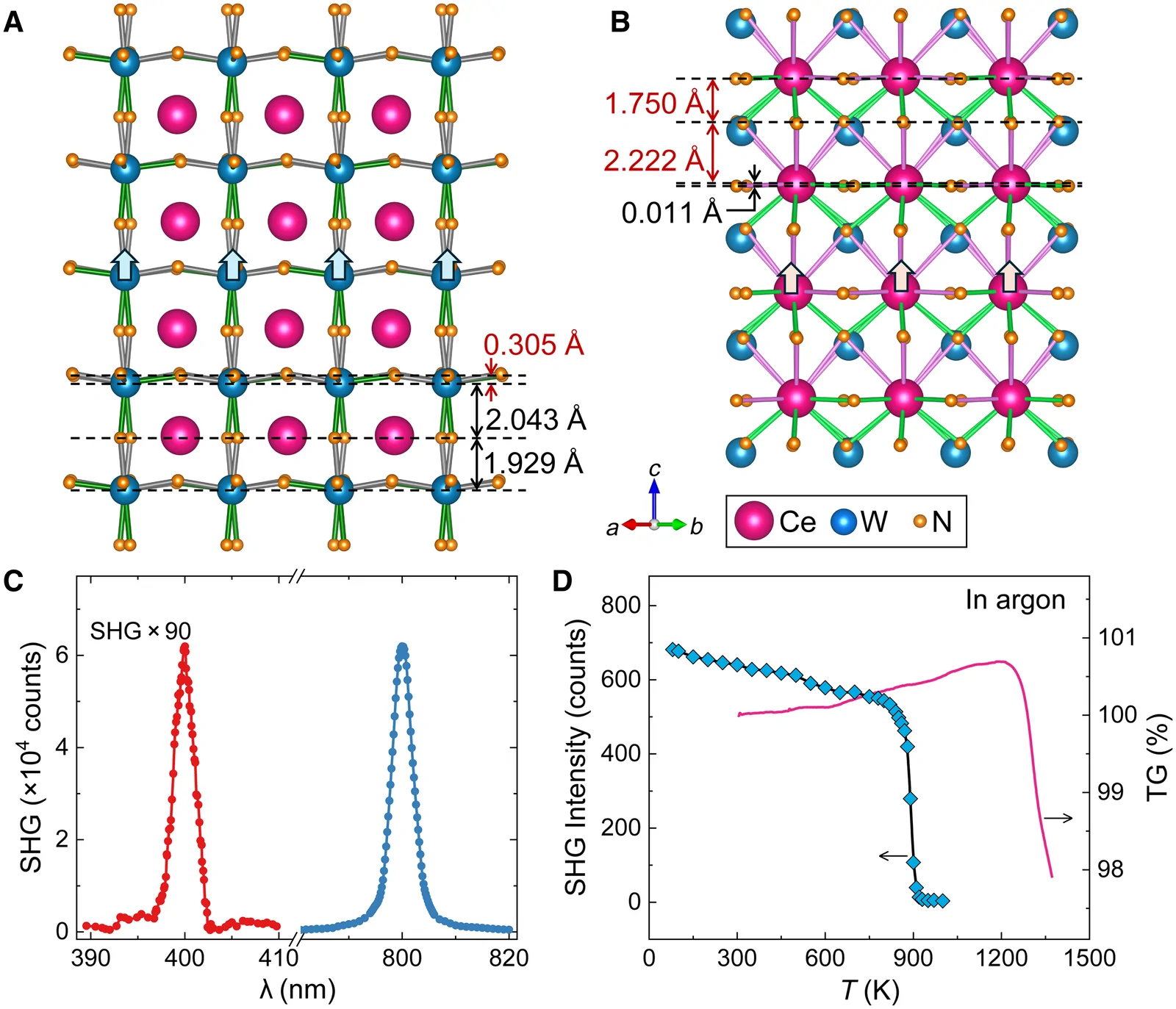
Polar origin and thermal stability of CeWN_3_. Schematic illustrations of polar distortions in (**A**) W and (**B**) Ce sublattices ([110] projection; pseudocubic [100]_pc_). Bonds are bisected by lengths: Gray (*d*_W─N_ < 2.05 Å) and green (*d*_Ce─N_ < 2.87 Å) mark the shorter half; olive (*d*_W─N_ > 2.05 Å) and pink (*d*_Ce─N_ > 2.87 Å) mark the longer half. Numerical labels mark the displacement magnitudes of cations relative to the N charge centers; red and black numbers represent displacements oriented parallel and antiparallel to the *c* axis, respectively. Thick arrows denote the resulting polarization directions for the two sublattices. (**C**) Second harmonic generation (SHG) signal acquired at 400 nm at RT. (**D**) Temperature-dependent SHG intensity (left) and thermogravimetric analysis (TGA; right) measured in flowing argon.

In the polar *Pna*2_1_-symmetric CeWN_3_, the refined atomic positions enable a quantitative evaluation of the magnitude of polarization arising from cation off-centering. As shown in Fig. 2 (A and B) and fig. S7, cation-anion layers stack along the *c* axis with alternating long and short interlayer spacings, indicative of periodic asymmetric structural modulation. Within the layers, W and Ce are displaced from the in-plane N coordination centers by +0.305 and −0.011 Å, respectively, while additional displacements of +0.472 Å (Ce) and −0.114 Å (W) along the *c* axis further enhance the polarity. These collective off-center distortions break inversion symmetry and generate a net polarization strictly along the *c* axis, which is the unique polar direction of the *Pna*2_1_ phase. By contrast, the in-plane displacements are symmetry compensated by glide-plane operations, resulting in zero polarization within the *ab* plane. Using a point-charge model, the spontaneous polarization is estimated as *P*_s_ = 32.6 μC/cm^2^ for the current CeWN_3_, dominated by the large formal charge and pronounced off-centering of the W^6+^ ions.

To further determine the polarity of CeWN_3_, temperature-dependent second harmonic generation (SHG) measurements were performed in argon. As shown in Fig. 2C, excitation with an 800-nm pulsed laser can generate intense SHG signals at around 400 nm at RT, in good agreement with the polar structure of CeWN_3_ mentioned previously. Additional laser-power–dependent measurements show a quadratic dependence of the SHG intensity on the incident laser power, whereas excitation-wavelength–dependent measurements confirm that the detected signal appears at half of the incident wavelength (fig. S8). Moreover, upon heating, the SHG signal remains robust up to ∼850 K and then decreases sharply to zero at ∼900 K (Fig. 2D). Thermogravimetric analysis (TGA) reveals that the thermal stability of CeWN_3_ is closely related to atmosphere. The sample remains chemically stable up to 1200 K in flowing argon (Fig. 2D), whereas a clear thermal anomaly occurs at 500 K in air (fig. S9). Temperature-dependent SXRD measurements in air-sealed capillaries further reveal a structural evolution from the initial polar *Pna*2_1_ phase to a high-temperature centrosymmetric *Cmmm* structure with a halved *c* axis. The *Pna*2_1_ and *Cmmm* phases coexist between ∼450 and 700 K (Supplementary Text and figs. S10 and S11).

### Metallic transport properties

**Fig. 3.**
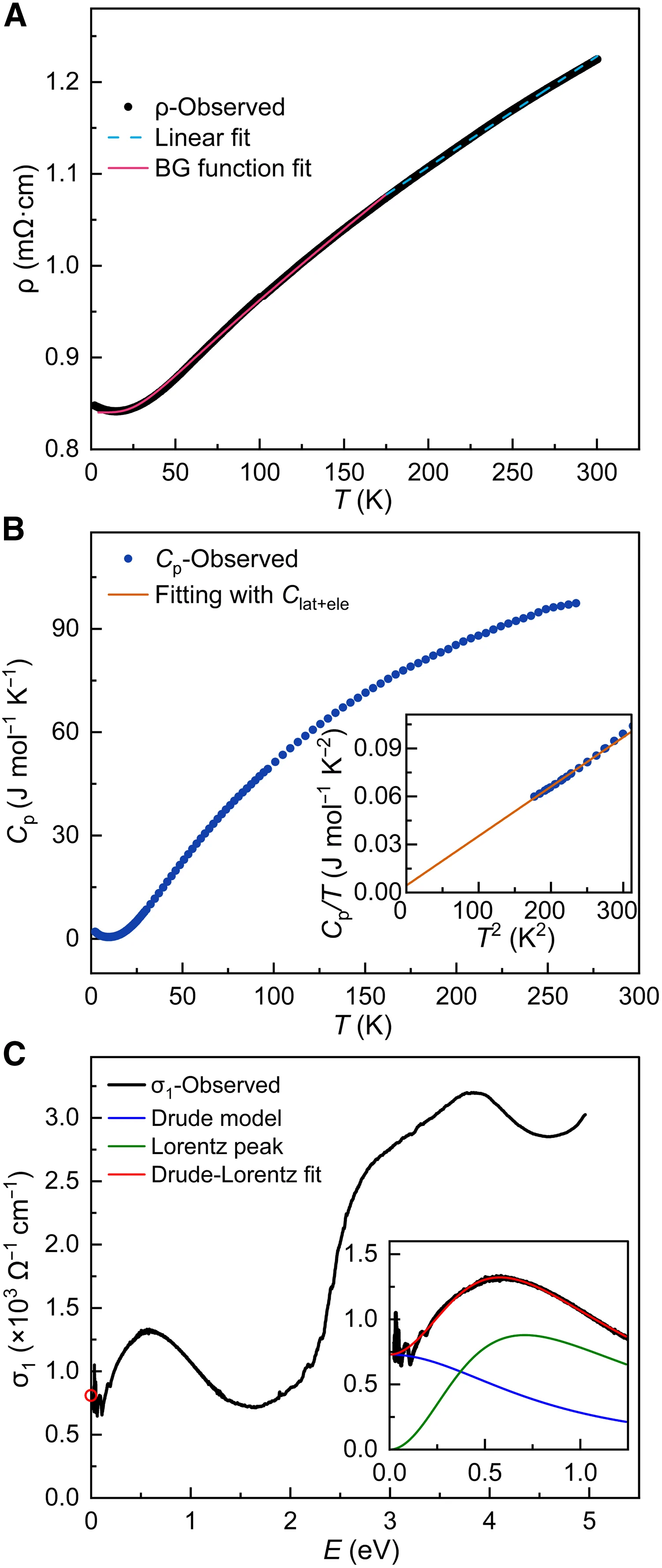
Metallic electrical transport properties of CeWN_3_. Temperature-dependent (**A**) electrical resistivity and (**B**) heat capacity. Inset in (B) presents *C*_p_/*T* versus *T*^2^ plot and linear fit incorporating phonon and electron contributions. (**C**) Optical conductivity measured at RT. The red circle on the vertical axis denotes the dc conductivity extracted from resistivity measurement. The inset shows Drude-Lorentz fitting results.

Figure 3A shows the electrical resistivity of CeWN_3_ as a function of temperature, measured from 300 to 2 K. The resistivity monotonically decreases from 1.22 to 0.84 mΩ·cm upon cooling with a positive temperature coefficient (*d*ρ*/dT* > 0), suggesting the metallic conducting nature. At higher temperatures (175 to 300 K), the resistivity exhibits a linear dependence on temperature, indicating dominant electron-phonon scattering, which is a hallmark of conventional metallic transport. A linear fitting using the function ρ(*T*) = ρ_0_ + α*T* yields a residual resistivity of ρ_0_ = 0.86 mΩ·cm and temperature coefficient of α = 1.21 μΩ·cm K^−1^, both comparable with those of classical binary metallic nitrides as observed in CeN and WN (*49*, *50*). However, below 150 K, the resistivity deviates from linearity and can be described by the Bloch-Grüneisen (BG) function, $\rho \left(T\right)=\rho_{0}^{\prime }+A{\left(T/\Theta_{D}\right)}^{5}\int_{0}^{\Theta_{D}/T}\frac{x^{5}}{\left(e^{x}-1\right)\left(1-e^{-x}\right)}\mathrm{dx}$, which accounts for the electron-phonon scattering weighted by the Debye phonon spectrum. The fitting yields $\rho_{0}^{\prime }$ = 0.84 mΩ·cm, a prefactor *A* = 1.01 mΩ·cm, and an effective Debye temperature $\Theta_{D}$ = 173.7 K. These results confirm the intrinsic metallic behavior of CeWN_3_ with well-characterized electron-phonon scattering features.

Furthermore, the itinerant electronic behavior of CeWN_3_ is revealed by heat capacity measurements. As shown in Fig. 3B, the specific heat (*C*_p_) gradually decreases from 300 to 5 K, which is consistent with the phonon population effects described by the Debye model. Furthermore, the *C*_p_ data in the linear region, excluding the low-temperature magnetic/Schottky-like contribution discussed in the Supplementary Text and fig. S12A, can be fitted by *C*_p_/*T* = γ + β*T*^2^, yielding γ = 2.7(8) mJ mol^−1^ K^−2^ and β = 0.33(1) mJ mol^−1^ K^−4^, owing to the electronic and lattice contributions, respectively. The Debye temperature derived from β is Θ_D_ = 181 K, which is similar to the value obtained from BG fitting of the resistivity (173.7 K). The moderate Sommerfeld coefficient γ provides additional evidence for itinerant carriers near the Fermi level. Notably, neither the heat capacity nor the magnetization measurements show any long-range spin order occurring in CeWN_3_ above 2 K, whereas some traces imply the presence of an antiferromagnetic (AFM) phase transition near 1 K, as discussed in the Supplementary Text and fig. S12B. A detailed investigation on the magnetism is interesting. However, it is beyond the scope of this study.

Optical conductivity measurements were performed to further elucidate the low-energy charge-carrier dynamics underlying the metallic state. As shown in Fig. 3C, the real part of the optical conductivity, σ_1_(ω), extrapolates to a substantial zero-frequency value of ∼800 Ω^−1^ cm^−1^, which is consistent with the dc conductivity (highlighted with a red circle) obtained from resistivity measurements, thereby corroborating the metallic transport nature of CeWN_3_. In the low-energy region below 1.2 eV, the optical response is effectively reproduced by a Drude-Lorentz model, as shown in the inset of Fig. 3C. The fitting yields a Drude plasma frequency of ω_p,D_ = 1.89 eV and a scattering rate of Γ_D_ = 0.62 eV. The presence of a strongly broadened Drude component indicates substantial carrier scattering, which is in agreement with the moderate residual resistivity observed in the transport measurements. Additionally, a broad mid-infrared absorption band centered at ∼0.6 eV suggests incoherent electronic excitations, likely associated with hybridization between itinerant W─N states and localized Ce 4f electrons. At higher energies, the absorption edge at 2 to 3 eV and the pronounced peak near 3.8 eV can be attributed to the interband transitions governed by the detailed electronic band structure.

### Electronic structure and origin of polar metallic state

**Fig. 4.**
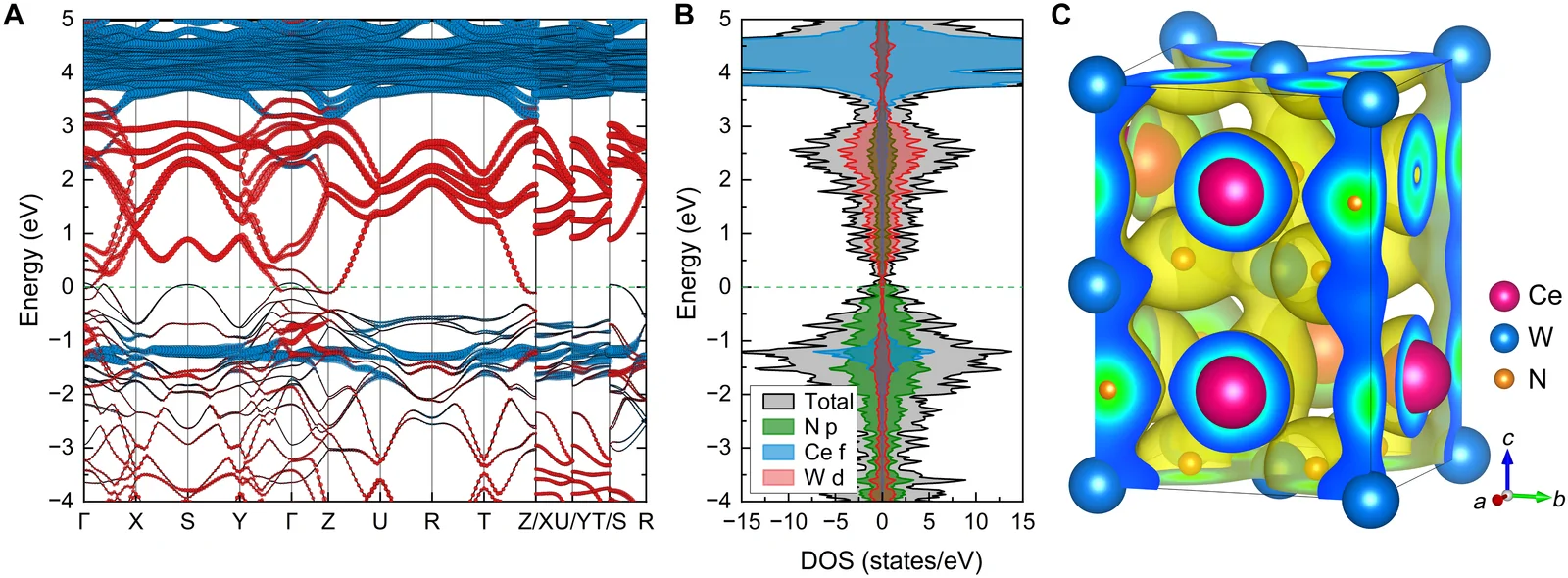
Electronic band structure of CeWN_3_ from first-principles calculations. (**A**) Projected band structure with Ce (blue) and W (red) orbital contributions. (**B**) Corresponding atom-resolved partial density of states. (**C**) Three-dimensional charge density distribution, plotted at an isovalue of ±0.05 *e* Å^−3^.

First-principles calculations were performed to explore the ground-state electronic properties of CeWN_3_ with a polar *Pna*2_1_ symmetry. Several magnetic configurations of Ce^3+^ ions, including nonmagnetic, ferromagnetic, and A-, C-, and G-type AFM orders, were examined. All spin configurations converge to an A-type AFM ground state, where Ce carries a local moment close to 1.0 μ_B_, while the magnetic contributions from W and N are negligible, as expected from the Ce^3+^/W^6+^ charge combination. The spin-polarized charge distribution (fig. S13) demonstrates that the occupied Ce 4f states are highly localized with minimal hybridization with W and N, retaining an essentially atomic-like character. The calculated band structure and density of states of CeWN_3_ in the A-type AFM ground state are shown in Fig. 4 and fig. S14. The conduction bands, spanning 0 to 3.5 eV, are primarily derived from W 5d *t*_2g_ orbitals, and the valence bands between −3.5 and 0 eV are dominated by N 2p states. Strong hybridization between W 5d and N 2p states occurs near the Fermi level, leading to metallic (or semimetallic) band structural characteristics, particularly around the Γ point, in good agreement with the experimental results. By contrast, the localized Ce 4f states are fully embedded near −1.2 eV within the valence band and exhibit negligible hybridization with N. Moreover, changing the on-site Coulomb interactions for W (0 to 2 eV) and Ce (4 to 5 eV), in our attempts, does not alter the overall band dispersion and hybridization features.

The simulated real-space charge-density distribution (Fig. 4C) reveals a continuous and interconnected W─N electron cloud that forms the backbone of the conduction pathways, whereas the electron density associated with Ce is confined to isolated regions and remains spatially localized. This sharp contrast in the bonding behavior can be traced to the distinct local crystallographic environments. Ce resides in a relatively open coordinated shell with long Ce─N bonds, which limit 4f-2p overlap and result in an electronically underbonded configuration. In comparison, W is located at the center of a compact octahedron, forming short and covalent W─N bonds strengthened by the high formal valence and strong electronegativity difference with N. These features promote strong 5d-2p hybridization and facilitate itinerant electron behavior. Consequently, the pronounced bonding asymmetry in CeWN_3_ establishes a strongly distorted WN_6_ framework that enables the coexistence of metallic conductivity and polar order. These findings highlight CeWN_3_ as a polar metal in which polar displacements and itinerant carriers arise predominantly from the WN_6_ octahedral sublattice. The structural stability of the WN_6_ octahedra in CeWN_3_ perovskite nitride provides a unique opportunity to realize a thermally stable polar metallic state at high temperatures.

Given the important role of nitrogen stoichiometry in perovskite nitrides, we further compare the present stoichiometric CeWN_3_ with previously reported LaWN_3−δ_ compounds. The reported LaWN_2.5_ thin-film (*23*) and bulk LaWN_3−δ_ samples (*31*) adopt polar structures and show semiconducting-like behavior. Moreover, the optical absorption edge reported for bulk LaWN_3−δ_ was obtained within a limited low-energy spectral range and may correspond to an interband absorption feature rather than fully excluding low-energy itinerant carriers. These observations suggest that LaWN_3−δ_ may lie close to a narrow-gap or semimetallic regime, with the transport behavior strongly affected by nitrogen vacancies, crystallinity, disorder, and grain-boundary effects. With respect to the current CeWN_3_, EDS measurements, XAS analysis, and TGA performed under an air atmosphere (fig. S9) confirm that the nitrogen content is very close to the chemically stoichiometric value. Therefore, the current CeWN_3_ exhibits metallic electrical transport while maintaining a similar polar *Pna*2_1_ structure. This behavior can be well understood from theoretical calculations, which indicate that strong W 5d–N 2p hybridization dominates the metallic transport. Full nitrogen stoichiometry effectively enhances this hybridization, favoring the emergence of metallic conductivity. Consequently, a polar-metal state, rather than a polar-semiconductor state, is realized in fully stoichiometric CeWN_3_.

In summary, a high-quality bulk CeWN_3_ perovskite nitride was successfully synthesized via high-pressure (5 GPa) and high-temperature (1773 K) solid-state reactions. By combining SXRD with different types of electron microscopy techniques, a polar orthorhombic *Pna*2_1_ structure was assigned to CeWN_3_. SHG measurements further confirmed the polar symmetry and demonstrated that the polar response persists up to 850 K in argon. Electrical transport, specific heat, and optical conductivity measurements revealed the metallic conducting feature of CeWN_3_. Density functional theory (DFT) calculations demonstrated a spin-polarized metallic ground state, in which strong W─N orbital hybridizations sustained polar distortions and metallic electrical conductivity. Compared with representative oxide polar metals such as LiOsO_3_, these findings establish CeWN_3_ as a record-high-temperature polar metal with coexisting structural polarity and metallicity. Therefore, the perovskite-structured nitride family with stoichiometric chemical composition offers a promising pathway for realizing thermodynamically stable polar metallic states well above RT, providing promising applications in fields such as noncentrosymmetric quantum transport and polar electronics.

## MATERIALS AND METHODS

### Material synthesis

High-purity powders of CeO_2_, WO_3_, and NaNH_2_ were weighed in a molar ratio of 1:1:7 and thoroughly ground in an argon-filled glove box. The mixture was sealed in platinum capsules (4 mm in diameter and length) and subjected to high-pressure and high-temperature treatment at 5 GPa and 1773 K for 30 min using a cubic anvil–type apparatus. The reaction products were rapidly quenched to RT, followed by gradual decompression over 3 hours. The recovered material was ground into powder, rinsed with deionized water to remove residual sodium and reaction byproducts, and subsequently dried. The resulting single-phase powder was pressed at 8 GPa for 10 min, yielding dense bulk samples suitable for physical property measurements. Notably, the synthesis product was sensitive to the chemical states of starting materials and the airtightness of the reaction assembly. In our repeated trials, several routes using CeN, elemental Ce/W, NaN_3_, and oxide/amide precursors could produce CeWN_3_ as the dominant phase. However, the oxide/amide route using CeO_2_, WO_3_, and NaNH_2_ provided better reproducibility, larger product yield, and lower required pressure. Routes using elemental metals or sodium azide generally required higher pressures, longer reaction times, or smaller reaction volumes and were more sensitive to the sealing quality of the reaction assembly. Therefore, the oxide/amide route was adopted for preparing the samples used in the structural and physical property measurements. Representative XRD patterns from different synthetic routes are shown in fig. S15.

### Crystal structure analysis

Phase purity and crystal structures were determined by powder XRD using a Huber diffractometer with Cu *K*_α_ radiation (λ = 1.5406 Å). RT SXRD measurements were performed using a large Debye-Scherrer camera installed at the BL02B2 beamline of SPring-8, Japan, with λ = 0.4205 Å. To examine the high-temperature structural evolution of CeWN_3_, temperature-dependent SXRD measurements were conducted from 100 to 1070 K in sealed capillaries using λ = 0.77534 Å. Rietveld refinement of the SXRD patterns was performed using the GSAS software package. TEM samples were prepared by dispersing CeWN_3_ powders in acetone and depositing the suspension onto a Mo holey-carbon TEM grid. SAED of CeWN_3_ was performed at RT along the [110] (pseudocubic [100]_pc_) and [001] (pseudocubic [001]_pc_) zone axes using a Tecnai G20 transmission electron microscope at an accelerating voltage of 200 kV. HAADF and MEP experiments were conducted at RT on a JEOL JEM-ARM300F2 scanning transmission electron microscope operating at 300 kV. The electron probe had a convergence semiangle of 24.3 mrad. In the MEP experiment, a defocused electron probe scanned the sample, and the diffraction patterns from all positions were combined into a 4D-STEM dataset comprising two real-space and two diffraction-space coordinates using a Gatan K3 camera. MEP reconstructions were performed using the robust mixed-state electron ptychography algorithms with multislice extension developed previously (*51*, *52*). The final MEP phase image projected along the [110] (pseudocubic [100]_pc_) axis of CeWN_3_ was obtained by summing the phase images along the depth dimension to visualize inequivalent nitrogen sites. STEM-NBED (4D-STEM) experiments were conducted at RT on an aberration-corrected FEI-Titan Cubed Themis 60-300 TEM operating at 300 kV. A probe-forming semiangle of 1.0 mrad was used. 4D-STEM NBED datasets were acquired using an EMPAD. The dataset covered a scanning area of 297 nm by 297 nm, with 200 × 200 uniformly distributed scanning points, and an exposure time of 15 ms per diffraction pattern. The beam current was set to 200 pA.

### Optical spectral characterization

SHG measurements at RT were conducted in a typical reflection geometry, with both the incident and reflected angles set to 45°. The incident laser beam was generated by a Ti:sapphire oscillator (Maitai, Spectra Physics) with a central wavelength of 800 nm (∼120-fs pulse duration, 82-MHz frequency). The incident beam was focused to a 20-μm-diameter spot on the sample surface. Laser-power–dependent SHG measurements were performed by varying the incident laser power, and excitation-wavelength–dependent measurements were conducted using incident wavelengths of 800, 900, and 1000 nm. Temperature-dependent SHG measurements were performed under flowing argon. For SHG measurements, the CeWN_3_ sample was polished to a thin slice using ultrafine diamond sandpaper. XAS measurements of the Ce-*L*_2,3_ edges were performed in total electron yield mode on beamline TLS 11A, whereas W-*L*_3_ edge spectra were measured in transmission mode on beamline TLS 17C at the National Synchrotron Radiation Research Center, Taiwan. Infrared reflectivity spectrum *R*(ω) was measured on a well-polished bulk disc specimen of CeWN_3_ with 2-mm diameter using a Bruker 80v Fourier transform infrared spectrometer within 0.02 to 1.5 eV. An in situ gold evaporation technique was used to obtain the absolute reflectivity of the sample. The reflectivity within the visible-ultraviolet range (1.25 to 5 eV) was measured at RT with an Avaspec 2048 × 14 optical fiber spectrometer. Then, Kramers-Kronig analysis of *R*(ω) was applied to obtain the optical conductivity spectra.

### Magnetic, electrical, and thermal measurements

Direct current magnetic susceptibility was measured using a magnetic property measurement system (MPMS3, Quantum Design). Zero-field-cooled and field-cooled data were collected. Temperature-dependent electrical resistivity (2 to 300 K) was measured using the standard four-probe method on a physical property measurement system (PPMS-9 T, Quantum Design). Electrical contacts were made with silver paste and gold wires on a bar-shaped pellet (∼2 mm by 1 mm by 1 mm), using a gauge current of 0.1 mA. Specific heat measurements were performed on the PPMS platform. TGA measurements were carried out on a Mettler TGA/DSC 3+ instrument under air and flowing argon atmospheres, using a heating rate of 5 K/min up to 1350 K.

### Theoretical calculations

First-principles calculations were carried out using DFT with the Vienna Ab initio Simulation Package. The calculations used the projector augmented wave method to describe the electron-ionic core interactions. After testing different electron correlation methods and onsite Coulomb interactions for the localized 4f orbitals, the strongly constrained and appropriately normed meta-generalized gradient approximation functional was chosen to describe the exchange-correlation interactions. For the Ce 4f orbitals, a Hubbard *U* of 4 to 5 eV was applied, whereas, for the W 5d orbitals, *U* ranging from 0 to 2 eV was tested. The wave functions were expanded in a plane-wave basis set with a cutoff energy of 700 eV. Forces on each ion were converged to less than 0.001 eV/Å, and a precision of 10^−8^ eV was used to minimize the total energy. A 7 × 7 × 5 *k*-mesh in the Monkhorst-Pack scheme was used for convergence in the self-consistent total energy calculations.
