# Supplementary material for: Observation of thermally stable polar metallic state in perovskite nitride CeWN3
Source: Sci Adv. 2026 Sep 25;12(39):eaeh6191. doi: 10.1126/sciadv.aeh6191 (PMC13614364; doi:10.1126/sciadv.aeh6191)
Supplement: Supplementary file 1 [file sciadv.aeh6191_sm.pdf]

Science Advances
AAAS

Supplementary Materials for

Observation of thermally stable polar metallic state in perovskite nitride $CeWN_3$

Xubin Ye *et al.*

Corresponding author: Zhen Chen, zhen.chen@iphy.ac.cn; Youwen Long, ywlong@iphy.ac.cn

Sci. Adv. **12**, eaeh6191 (2026)
DOI: 10.1126/sciadv.aeh6191

This PDF file includes:

Supplementary Text
Figs. S1 to S15
Tables S1 and S2

Supplementary Text

XAS measurements

XAS measurements at the Ce-$M_{4,5}$ and W-L_3 edges were carried out to determine the oxidation states of cations in $CeWN_3$. As shown in Supplementary Fig. S6A, the Ce-$M_{4,5}$ edges of $CeWN_3$ closely resemble those of the Ce^{3+} ($4f^1$) reference compound Ce_2O_3, exhibiting a characteristic multiplet structure with sharp and well-resolved M_4 and M_5 peaks, distinct low-energy shoulders, and notably lacking the satellite feature near 889 eV that is typical of Ce^{4+} species. The W-L_3 edge spectra (Supplementary Fig. S6B) display a symmetric intense white-line peak centered at 10215 eV, matching the W^{6+} reference WO_3. Importantly, no spectral broadening or low-energy shoulders, which are usually associated with partially reduced W species (e.g., W^{5+} or W^{4+}), are observed. Therefore, these results confirm a well-defined Ce^{3+}/W^{6+} charge configuration in $CeWN_3$, indicative of fully occupied nitrogen sites.

Temperature-dependent SXRD measurements

Temperature-dependent SXRD measurements were performed from 100 to 1070 K in air-sealed capillaries to examine the high-temperature structural evolution of $CeWN_3$. As shown in Supplementary Fig. S10A, the full-pattern evolution shows no obvious impurity formation upon heating, indicating that the perovskite-related phase is retained during the measurement. In the representative high-intensity reflection region (Supplementary Fig. S10B), the peak profile evolves from a two-peak feature at low temperatures to an apparent three-peak feature between approximately 450 and 700 K, and then returns to a two-peak feature at higher temperatures. This behavior suggests a two-phase region with coexistence of low- and high-temperature diffraction components. More pronounced changes are observed for selected weak reflections and superstructure peaks (Supplementary Fig. S10C). In the RT $Pna2_1$ indexing, the reflections that weaken or disappear upon heating mainly satisfy the condition $h + k + l = 2n + 1$, including (111), (201), (113), (221), (203), (131), (311), (133), (313), (115), and (331). The disappearance of these reflections suggests the suppression of the low-temperature structural modulation associated with the doubled c axis, making the high-temperature structure consistent with the halved c-axis $Cmmm$ phase.

Rietveld refinements of the temperature-dependent SXRD data further clarify the structural evolution (Supplementary Fig. S11). The low-temperature diffraction patterns are well described by the polar $Pna2_1$ structure. Upon heating, an additional *Cmmm* phase appears, giving rise to a $Pna2_1$ + *Cmmm* two-phase region between approximately 450 and 700 K. At higher temperatures, the diffraction patterns can be described by a single *Cmmm* phase. Compared with the low-temperature $Pna2_1$ setting, the *Cmmm* phase has a halved *c* axis, consistent with the disappearance of the weak/superstructure reflections associated with the low-temperature structural modulation. For comparison, the *c*-axis parameter and unit-cell volume of the *Cmmm* phase are doubled in Supplementary Fig. S11. Since *Cmmm* is centrosymmetric, the $Pna2_1$-to-*Cmmm* evolution is consistent with the suppression of polar distortion inferred from the disappearance of the SHG signal at elevated temperatures.

The characteristic temperature range observed by SXRD is close to the thermal anomaly observed by TGA under air atmosphere, whereas SHG measurements were performed under flowing argon. This difference is likely related to the distinct measurement environments. In argon, $CeWN_3$ remains chemically stable up to 1200 K and retains a clear SHG response up to approximately 850 K. In contrast, the SXRD measurements were conducted in air-sealed capillaries, where the structural evolution occurs at relatively lower temperatures, consistent with the atmosphere-sensitive high-temperature behavior revealed by TGA. These results indicate that the high-temperature structural evolution of $CeWN_3$ is sensitive to atmosphere, sealing condition, and sample environment.

Low-temperature magnetic properties

To further elucidate the low-temperature electronic and magnetic behavior of $CeWN_3$, heat-capacity and magnetic-susceptibility measurements were performed down to the sub-kelvin regime. As shown in Supplementary Fig. S12A, the heat capacity exhibits a broad anomaly centered around 2 K under zero magnetic field. With increasing external magnetic field, this feature shifts systematically to higher temperatures, a behavior characteristic of a Schottky-like contribution associated with the Zeeman splitting of the Ce^{3+} 4*f* crystal-field levels. This assignment is consistent with the highly localized nature of the Ce 4*f* electrons revealed by first-principles calculations and real-space charge-density analysis. In addition to the broad Schottky-like feature, a weaker but distinct peak (black arrow) appears near 1 K under zero field. This

anomaly is rapidly suppressed by external magnetic fields of 4 T and 6 T, indicating a magnetic origin and suggesting the emergence of long-range ordering of the localized Ce^{3+} 4*f* moments at very low temperatures.

The magnetic susceptibility data provide independent evidence for possible spin order near 1 K. As shown in Supplementary Fig. S12B, the ZFC and FC susceptibilities split below ~1 K, accompanied by a cusp-like feature around 0.6 K (inset), suggesting the occurrence of an antiferromagnetic order. At higher temperatures ($T > 75$ K), the susceptibility follows a modified Curie−Weiss behavior, $\chi = \chi_0 + C/(T-\theta)$. The extracted effective magnetic moment $\mu_{eff} = 2.25$ μ_B/f.u. is close to the theoretical value of 2.54 μ_B expected for a trivalent Ce^{3+} ($4f^1$), confirming the localized nature of the Ce 4*f* moments. The negative Weiss temperature $\theta = -20.4$ K indicates predominantly antiferromagnetic interactions among the Ce moments. Beyond the localized magnetic response, the sizable temperature-independent susceptibility term χ_0 points to a Pauli-like contribution from itinerant electrons. This observation is consistent with the metallic transport, finite Sommerfeld coefficient in the heat capacity, and optical conductivity results presented in the main text, and supports the coexistence of localized Ce 4*f* magnetism and itinerant carriers hosted by the W–N framework.

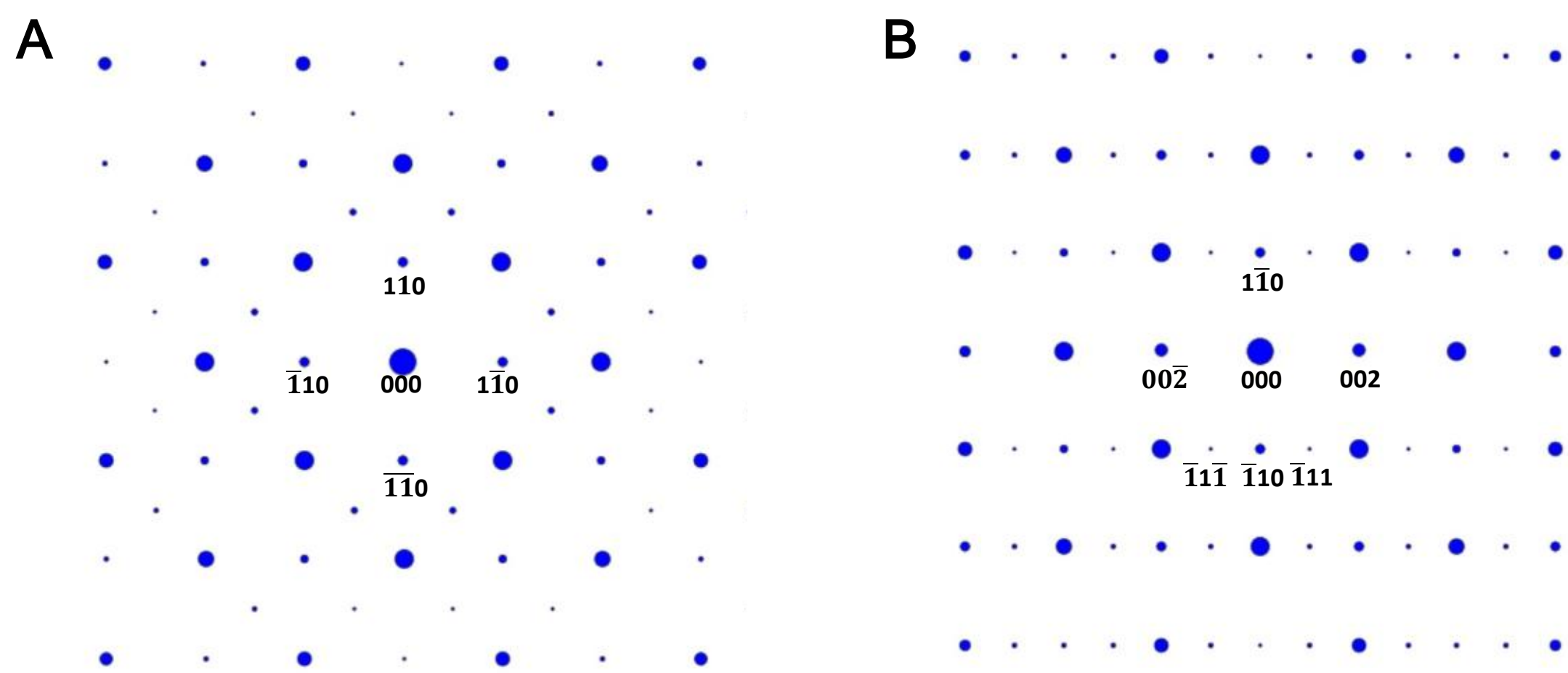

Fig. S1. Simulated SAED patterns along the (A) [001] (pseudo-cubic $[001]_{pc}$) and (B) [110] (pseudo-cubic $[100]_{pc}$) zone axes, based on the *Pna*2_1 structural model of $CeWN_3$.

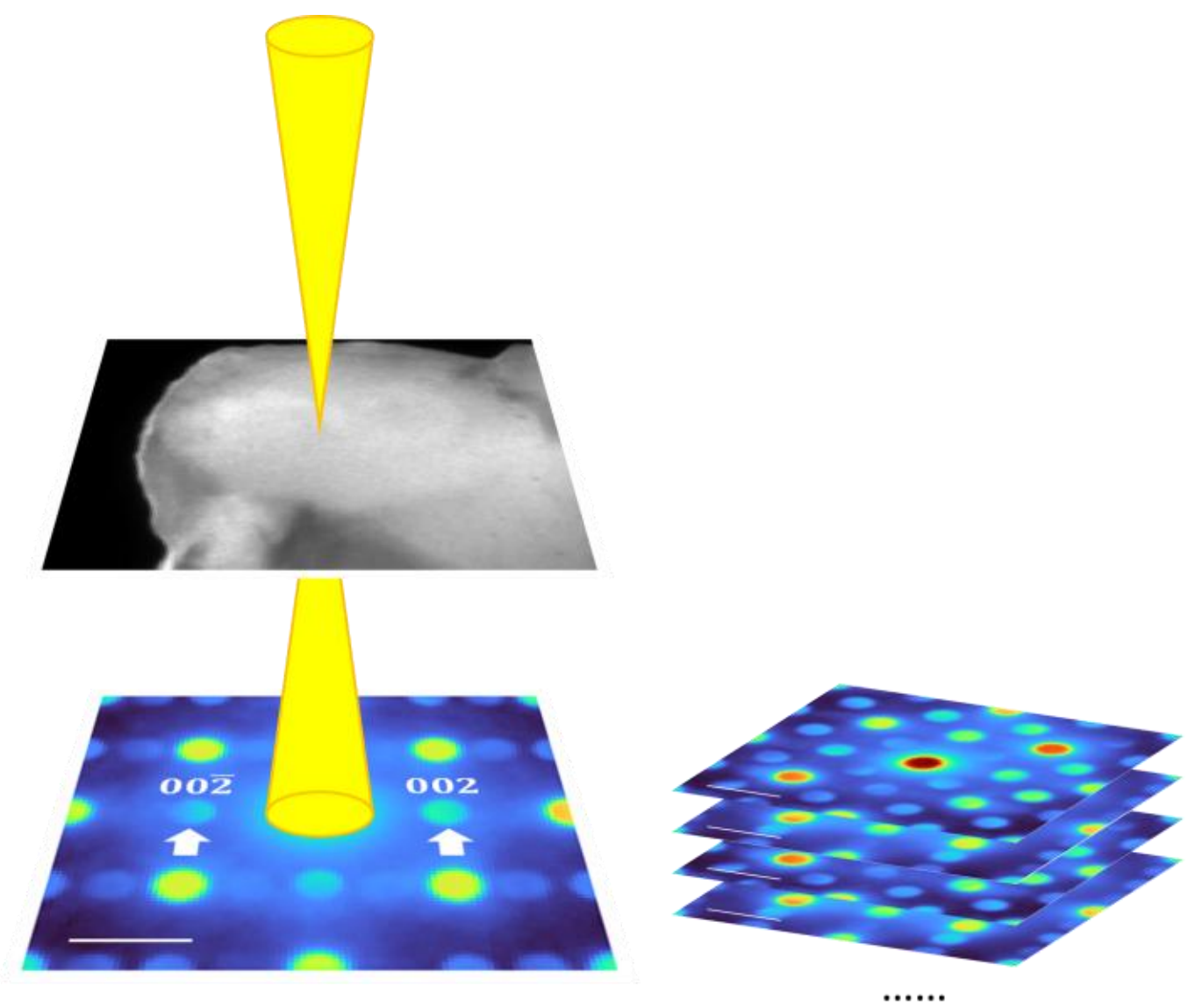

Fig. S2. Schematic of the STEM-NBED (4D-STEM) imaging technique on the sample flake, where a diffraction pattern was recorded at each probe position.

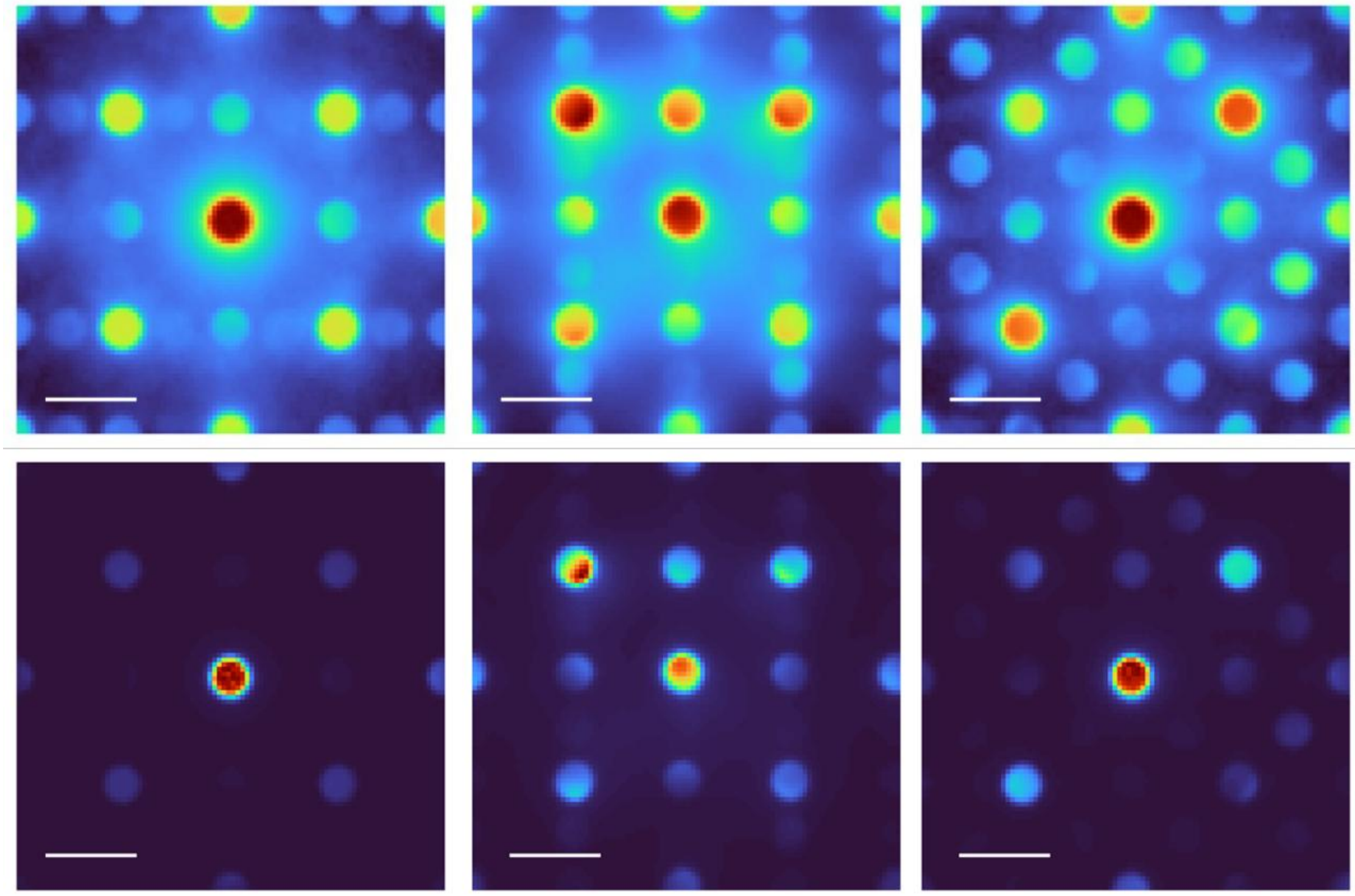

Fig. S3. NBED patterns of $CeWN_3$ acquired along the [110] (pseudo-cubic $[100]_{pc}$, left), $[1\bar{1}0]$ (pseudo-cubic $[010]_{pc}$, middle), and [001] (pseudo-cubic $[001]_{pc}$, right) zone axes, displayed using logarithmic (top) and linear (bottom) intensity scales.

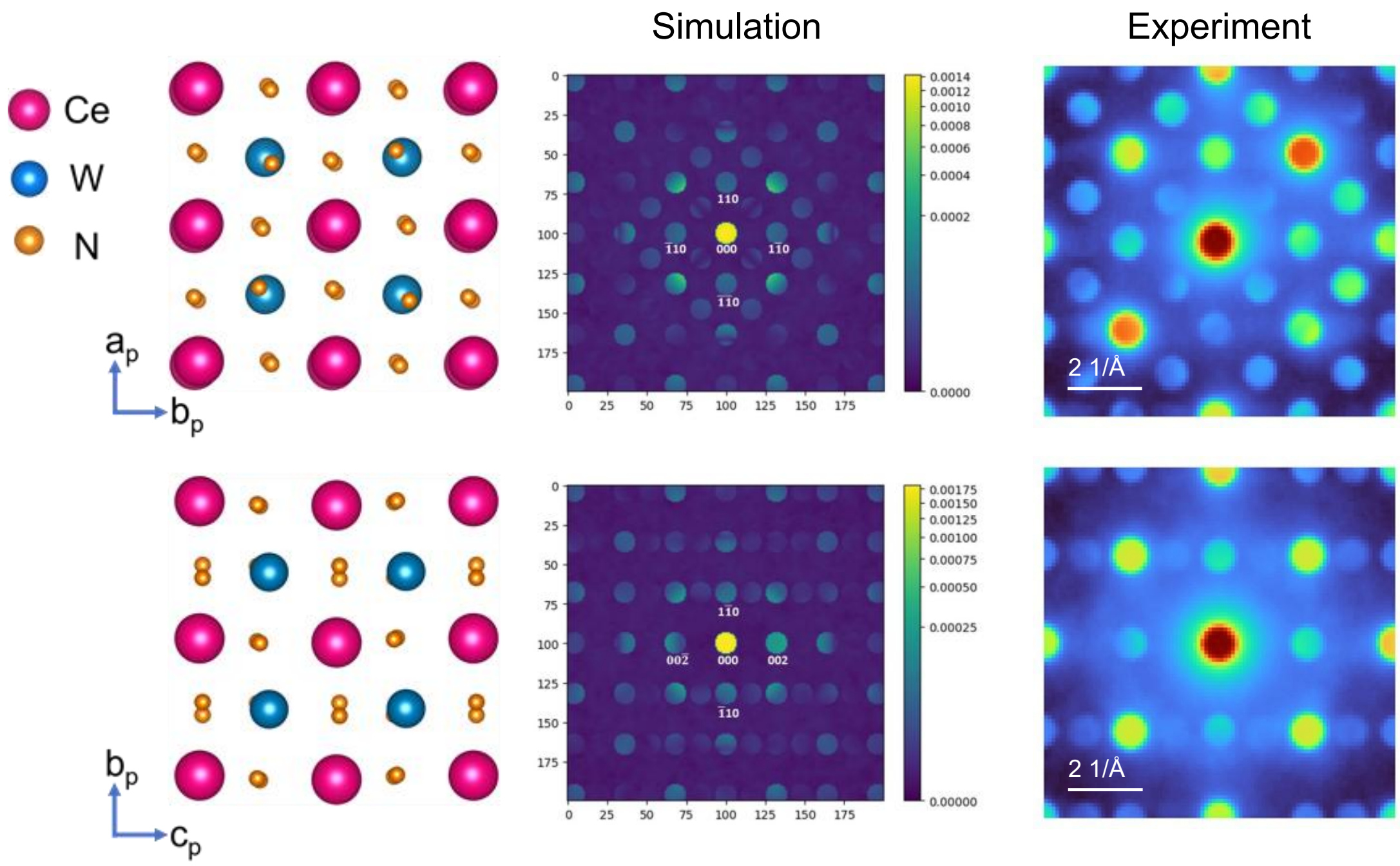

Fig. S4. Simulated (center) and experimental (right) NBED patterns of $CeWN_3$ along the [001] (pseudo-cubic $[001]_{pc}$, top) and [110] (pseudo-cubic $[100]_{pc}$, bottom) zone axes. In the top-right experimental pattern, a slight vertical beam misalignment (referenced by the central transmitted disk) introduces intensity asymmetry. By contrast, the bottom-right pattern exhibits negligible misalignment, and the observed asymmetry is attributed solely to the intrinsic lattice polarity.

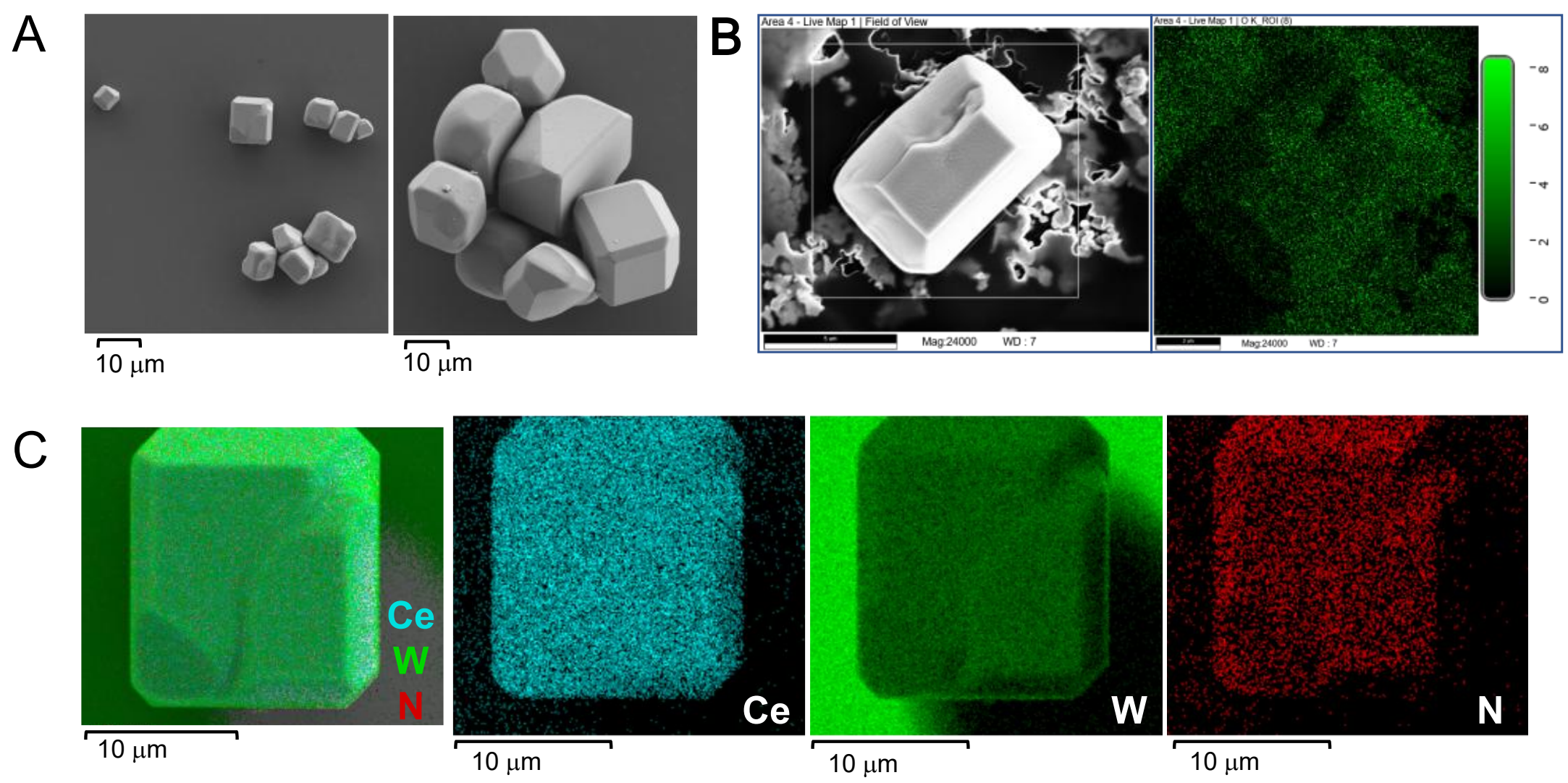

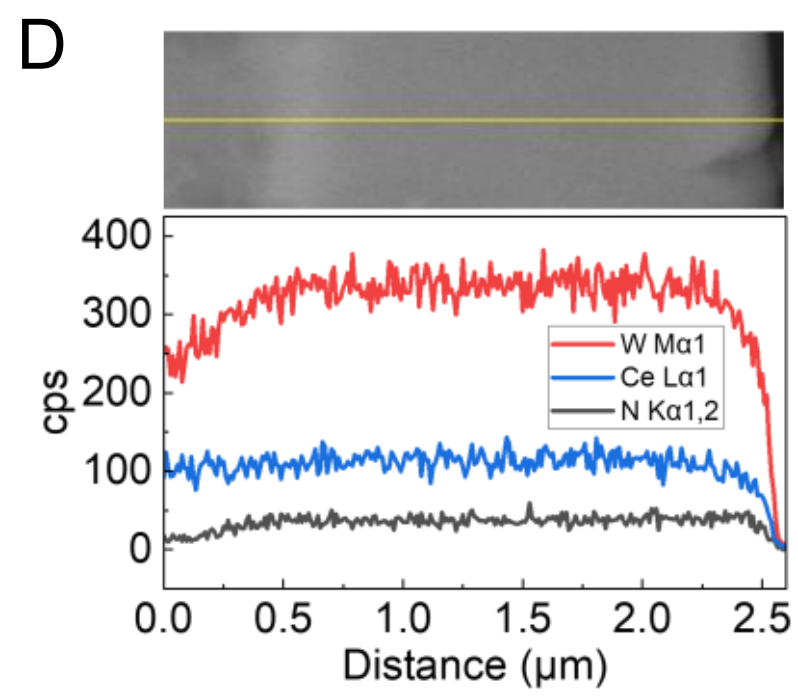

Element	Weight %	Weight % Sigma	Atomic %
N (K series)	11.42	0.23	59.81
Ce (L series)	38.75	0.24	20.30
W (M series)	49.83	0.25	19.89
Total	100		100

Fig. S5. SEM and EDS characterization of $CeWN_3$. (A) Representative SEM images showing well-faceted $CeWN_3$ microcrystals with typical sizes of ~10 µm, indicative of the high crystallinity achieved in the high-pressure-synthesized samples. (B) Representative O mapping acquired from a $CeWN_3$ crystal. The O mapping shows only a weak background-level signal and no obvious oxygen enrichment correlated with the crystal, indicating the absence of detectable oxygen-rich impurities within the detection limit of EDS mapping. (C) EDS elemental mapping of Ce, W, and N acquired from an individual crystal, revealing a homogeneous spatial distribution without detectable chemical segregation or compositional gradients. (D) EDS line-scan profiles across a representative crystal, together with quantitative elemental analysis. After correcting for surface topography effects, the elemental intensities remain nearly constant across the entire scan, confirming good compositional uniformity. The measured atomic percentages yield a normalized atomic ratio of Ce:W:N ≈ 1.00:0.98:2.95, close to the ideal stoichiometric ratio of 1:1:3 within experimental uncertainty.

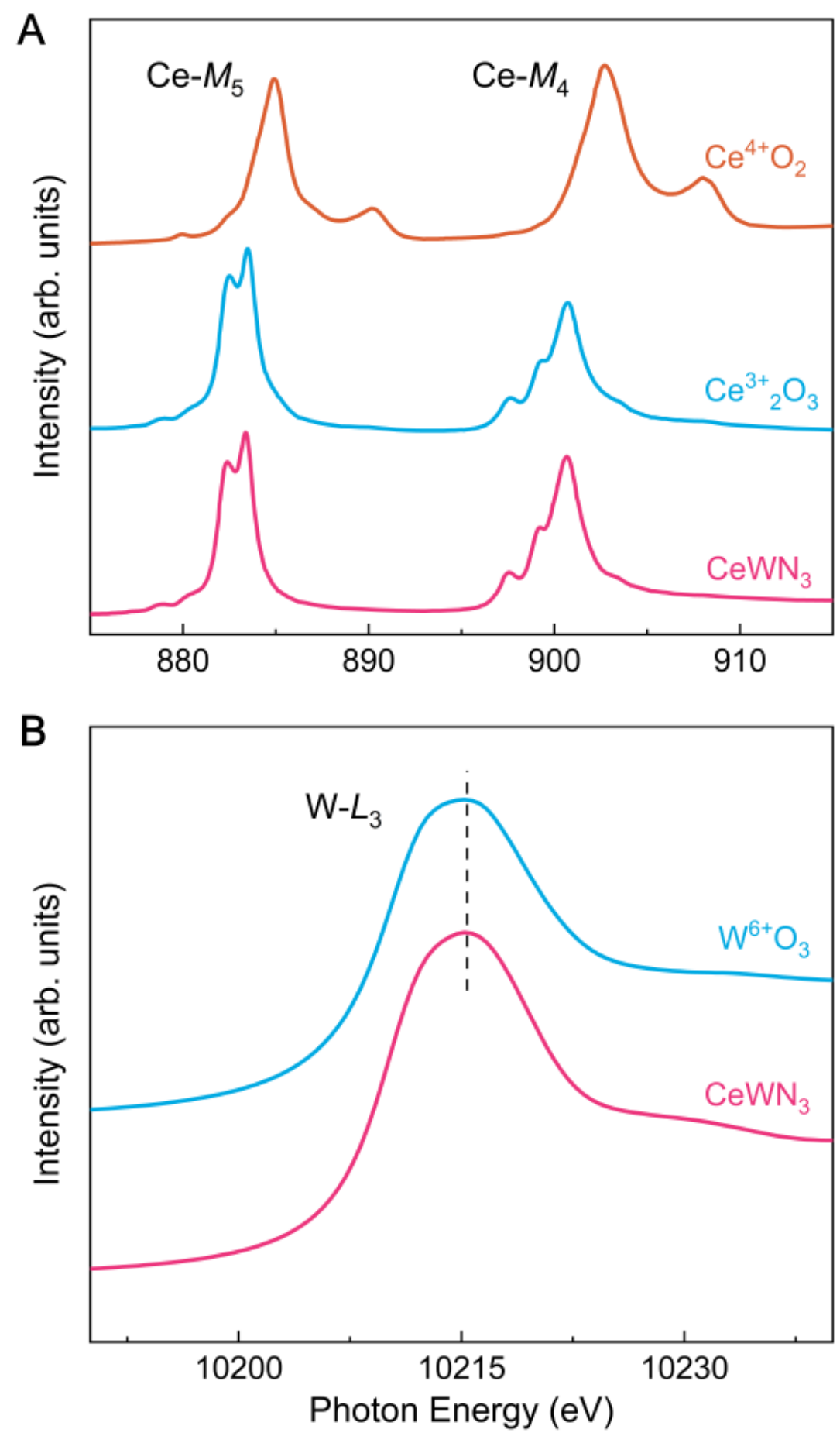

Fig. S6. XAS spectra of $CeWN_3$. (A) Ce-$M_{4,5}$ edges, and (B) W-L_3 edge. The references including Ce_2O_3, CeO_2, and WO_3 are presented for comparison.

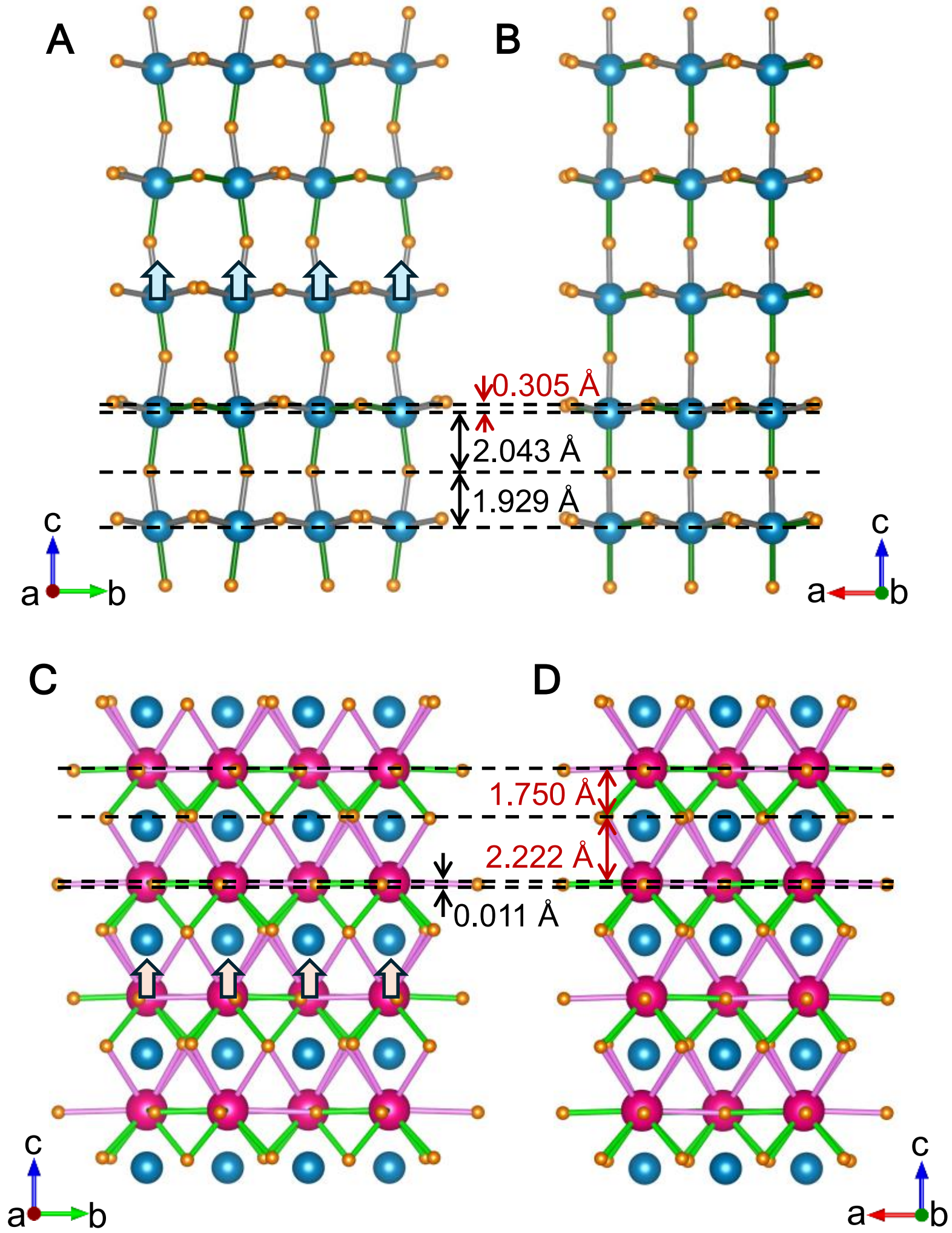

Fig. S7. Microscopic origin of polarization in $CeWN_3$. (A, B) W sublattice and (C, D) Ce sublattice distortions. The 2.05 Å (W–N) and 2.87 Å (Ce–N) thresholds divide the bonds into equal populations: shorter bonds (gray/green) and longer bonds (olive/pink). Views are projected along the *a*- and *b*-axes, respectively. Numerical labels mark the displacement of positive and negative charge centers along the *c* axis, with red numbers highlighting the dominant contributions to the net polarization.

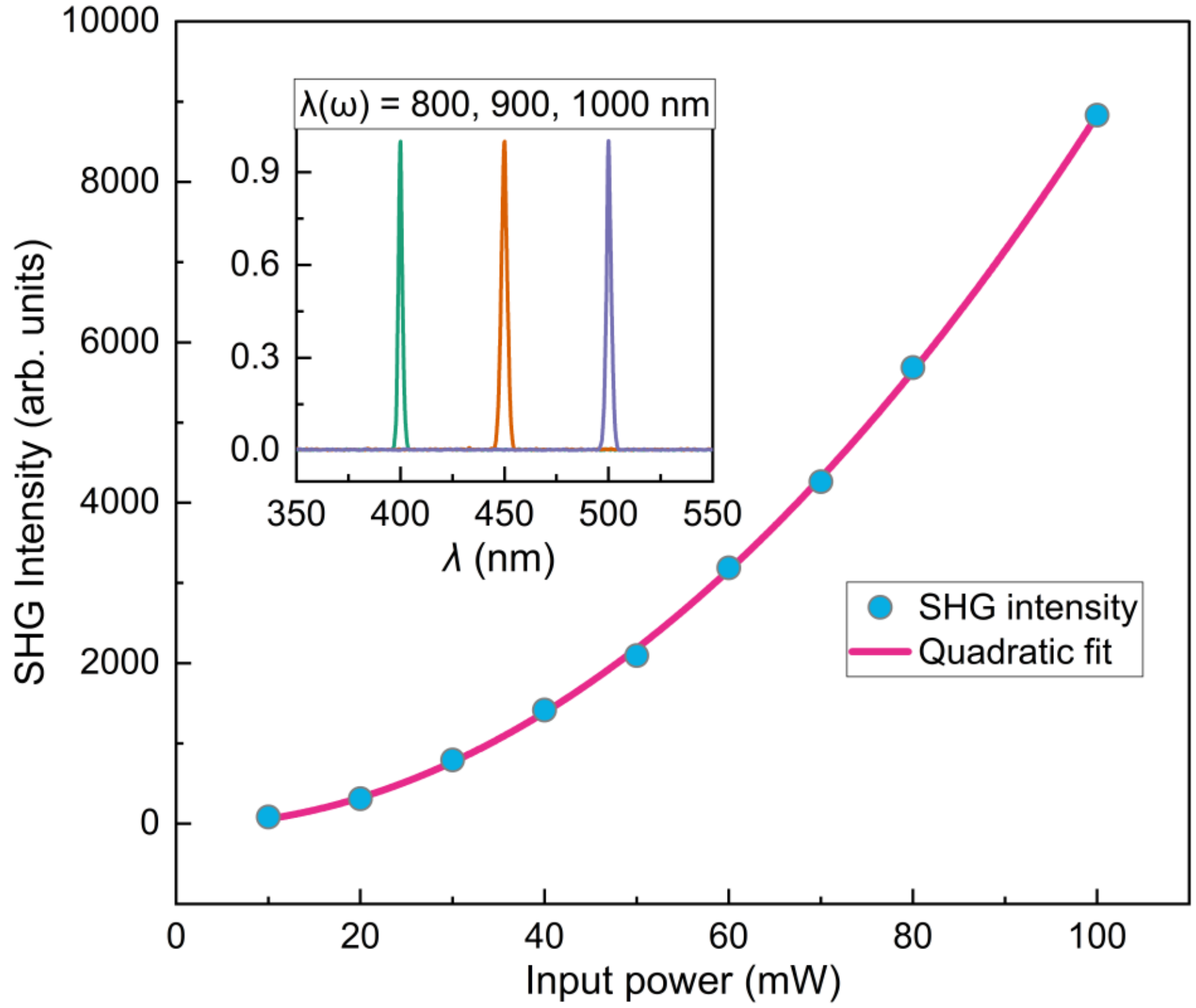

Fig. S8. Laser-power- and excitation-wavelength-dependent SHG measurements of $CeWN_3$ at room temperature. The SHG intensity exhibits a quadratic dependence on the incident laser power, confirming the second-order nonlinear optical nature of the observed signal. The inset shows SHG spectra measured with incident wavelengths of 800, 900, and 1000 nm, where the corresponding SHG peaks appear at 400, 450, and 500 nm, respectively.

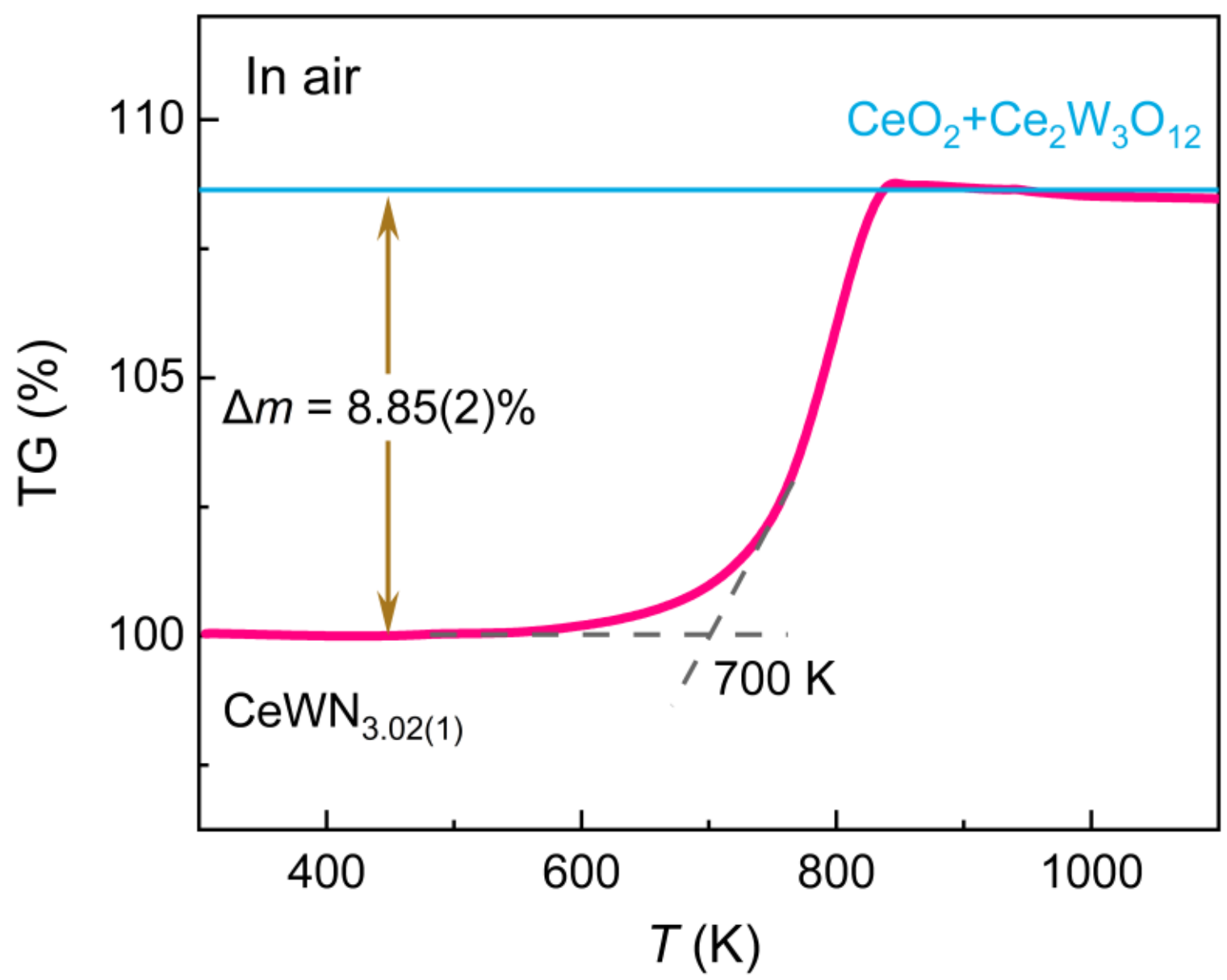

Fig. S9. TGA of $CeWN_3$ measured in air. Upon heating in air, $CeWN_3$ undergoes complete oxidative decomposition accompanied by a pronounced mass increase, yielding a yellow oxidation product. Laboratory XRD measurements identify the final residue as a 1:1 molar mixture of CeO_2 and $Ce_2W_3O_{12}$, providing a well-defined end state for quantitative compositional analysis. The total mass gain after full oxidation is 8.85(2)%, corresponding to an effective composition of $CeWN_{3.02(1)}$, which agrees well with the ideal stoichiometry of $CeWN_3$ within experimental uncertainty. This result provides independent evidence for the stoichiometric nitrogen content of the high-pressure-synthesized samples. For comparison, TGA measurements conducted under flowing argon (Fig. 2D) yield a black decomposition product composed of reduced Ce- and W-containing phases, including elemental species and substoichiometric oxides. The detailed decomposition pathway and mass evolution under inert atmosphere are highly sensitive to the sealing quality of the system and the purity of the argon gas, and therefore are not used for quantitative compositional analysis.

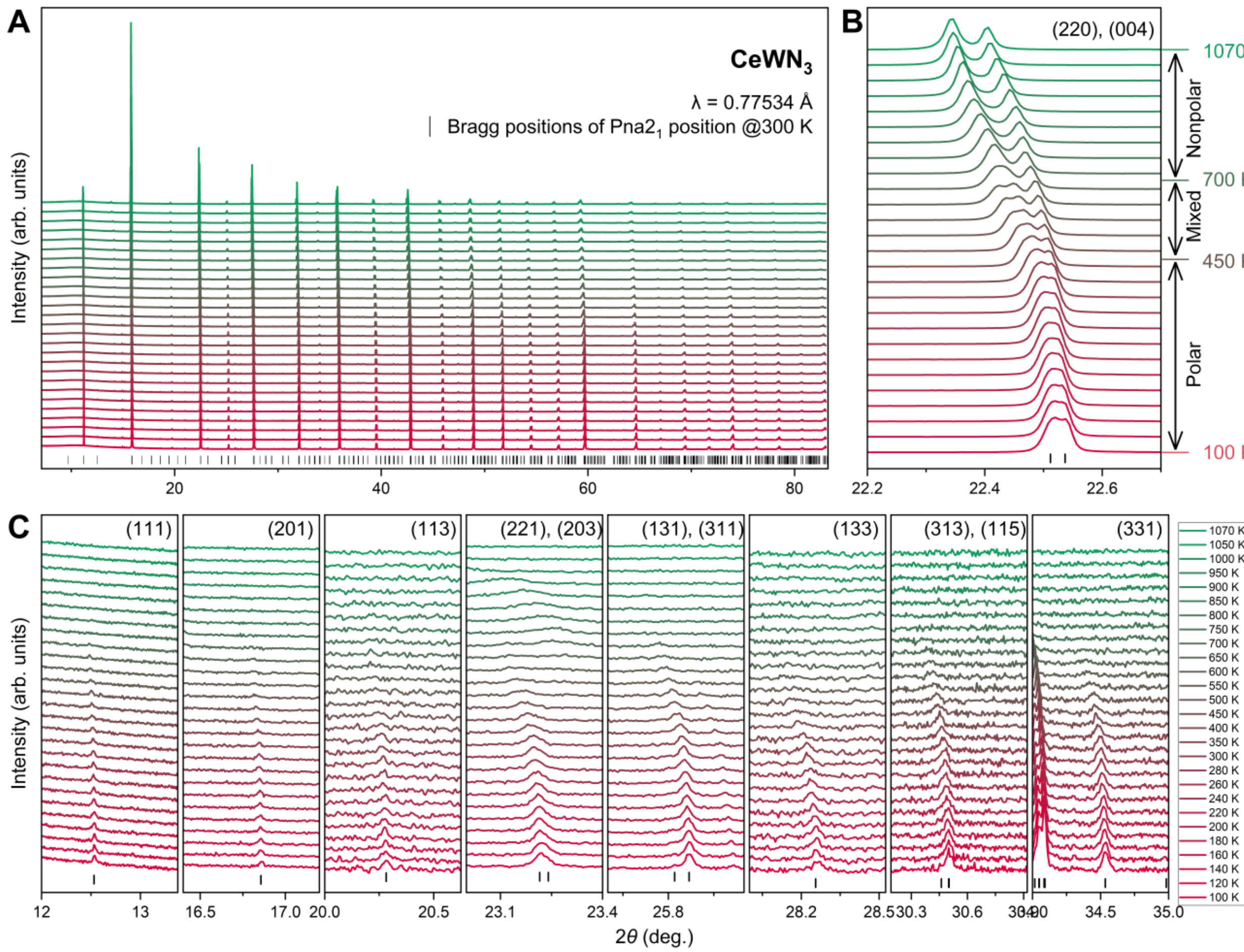

Fig. S10. Temperature-dependent SXRD patterns of $CeWN_3$ measured from 100 to 1070 K in air-sealed capillaries. (A) Stacked SXRD patterns collected between 100 and 1070 K. (B) Enlarged view of a representative high-intensity reflection region, showing the temperature evolution of peak splitting across the low-temperature polar, intermediate, and high-temperature regions. (C) Enlarged views of selected weak reflections and superstructure peaks. The gradual weakening and disappearance of these reflections upon heating clearly indicate a temperature-induced structural evolution of $CeWN_3$. The patterns are vertically offset for clarity. The (*hkl*) indices are indexed in the RT *Pna*2$_1$ setting. Black tick marks indicate the calculated Bragg positions of the polar *Pna*2$_1$ phase at 300 K.

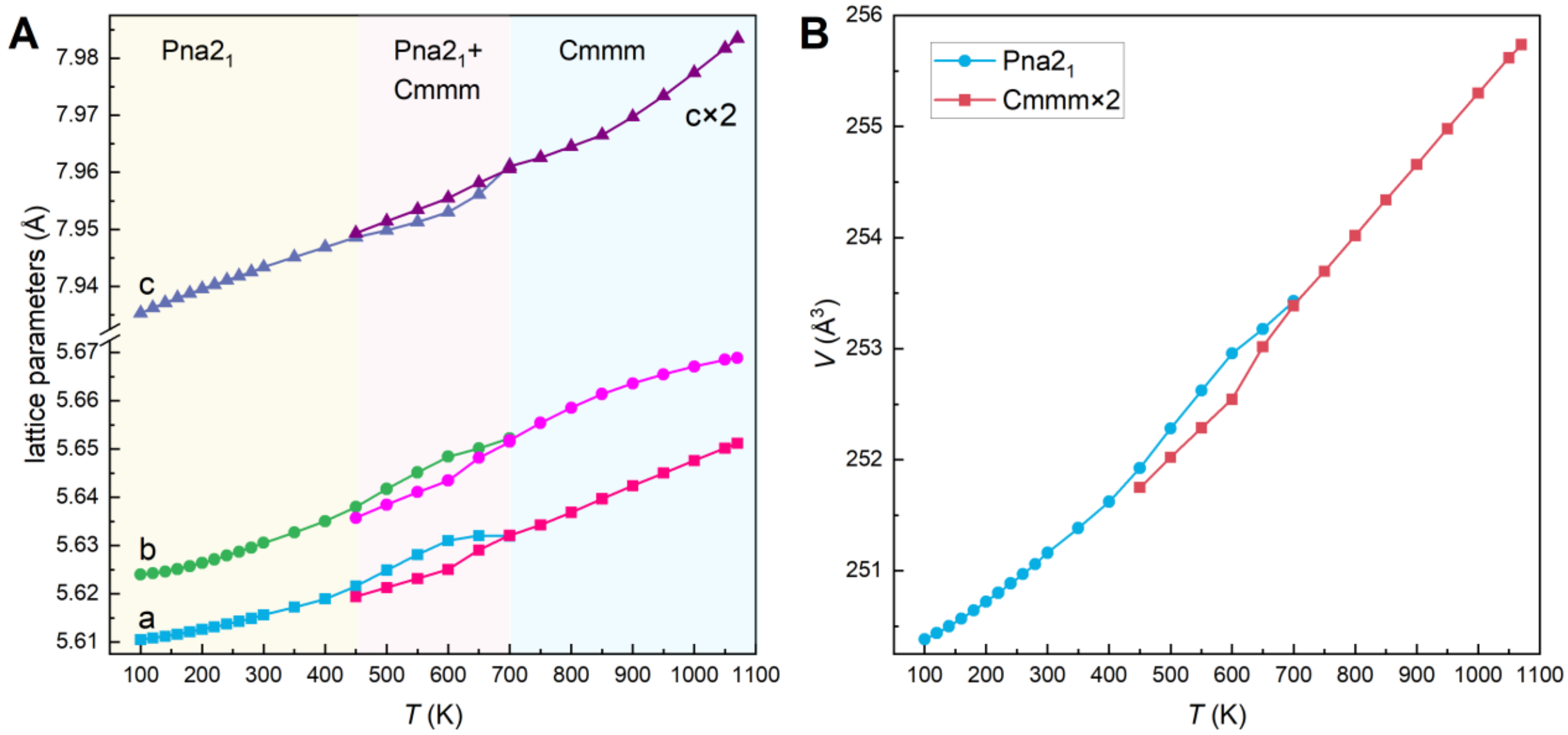

Fig. S11. Refined lattice parameters and unit-cell volume of $CeWN_3$ from temperature-dependent SXRD measurements. (A) Temperature evolution of lattice parameters. The low-temperature diffraction patterns are described by the polar *Pna*2$_1$ structure, while the high-temperature patterns are described by a centrosymmetric *Cmmm* structure. A *Pna*2$_1$ + *Cmmm* two-phase region is observed between approximately 450 and 700 K. The *c*-axis parameter of the *Cmmm* phase is doubled for comparison with the *Pna*2$_1$ setting. (B) Temperature evolution of unit-cell volume. The volume of the *Cmmm* phase is doubled for comparison with that of the *Pna*2$_1$ cell. Error bars are smaller than the symbols.

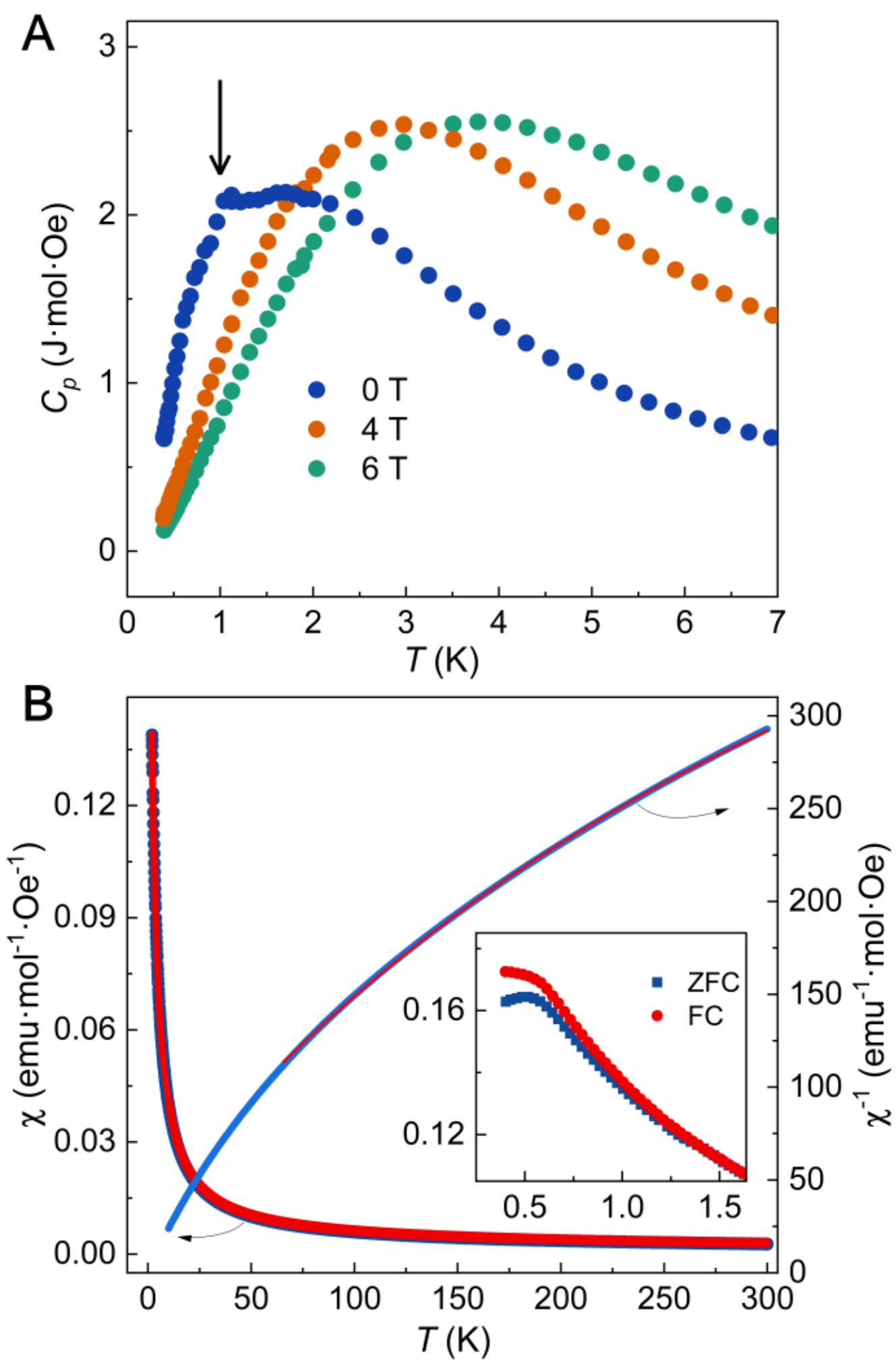

Fig. S12. Heat capacity and magnetic properties of $CeWN_3$. (A) The ultralow-temperature heat capacity under zero and applied magnetic fields. (B) Temperature dependence of magnetic susceptibility (left axis) and its inverse (right axis); the inset shows the extreme low temperature data.

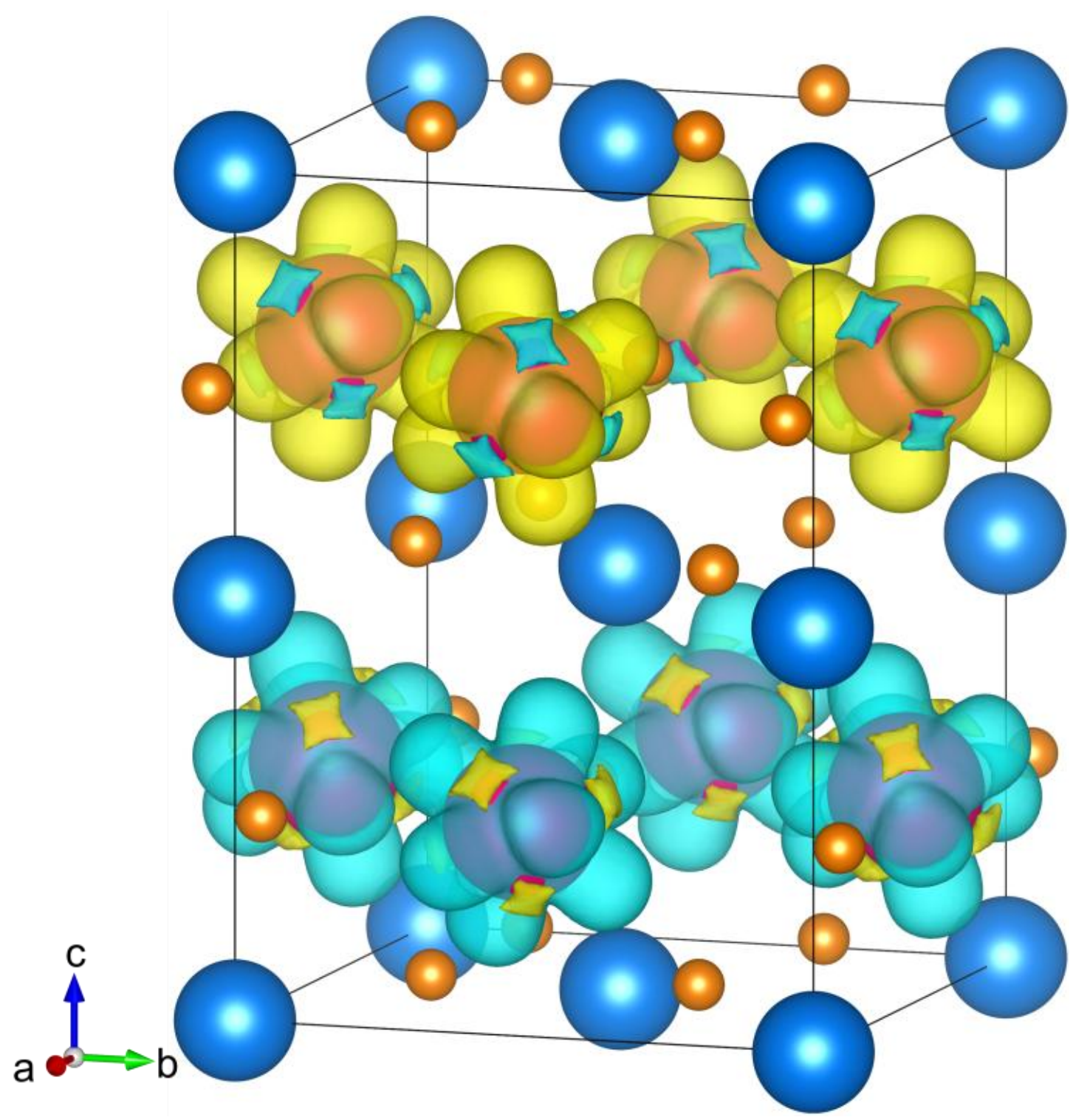

Fig. S13. Three-dimensional spin-polarized charge density distribution of $CeWN_3$. The yellow and cyan isosurfaces correspond to spin-up and spin-down magnetization densities, respectively, plotted at an isovalue of ±0.003 e·$Å^{-3}$.

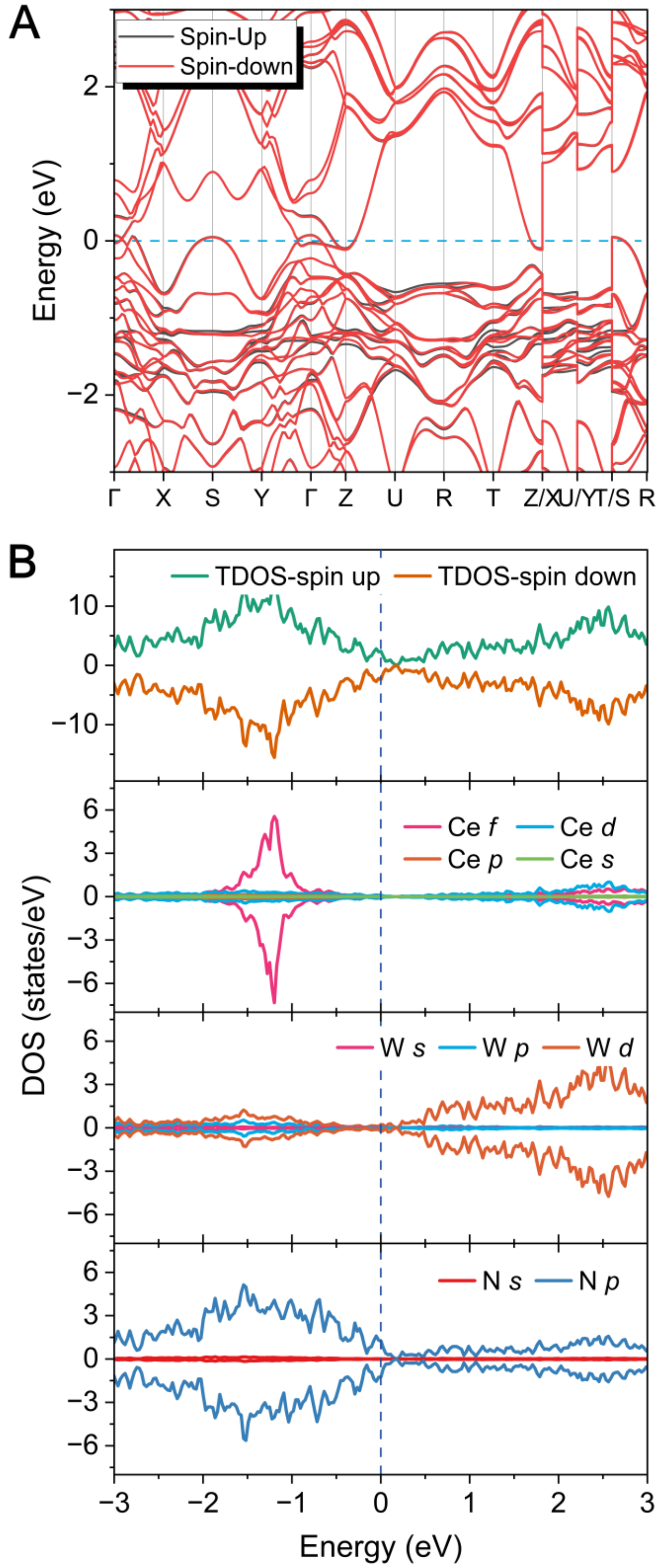

Fig. S14. Spin-resolved electronic band structure and orbital-resolved DOS of $CeWN_3$.

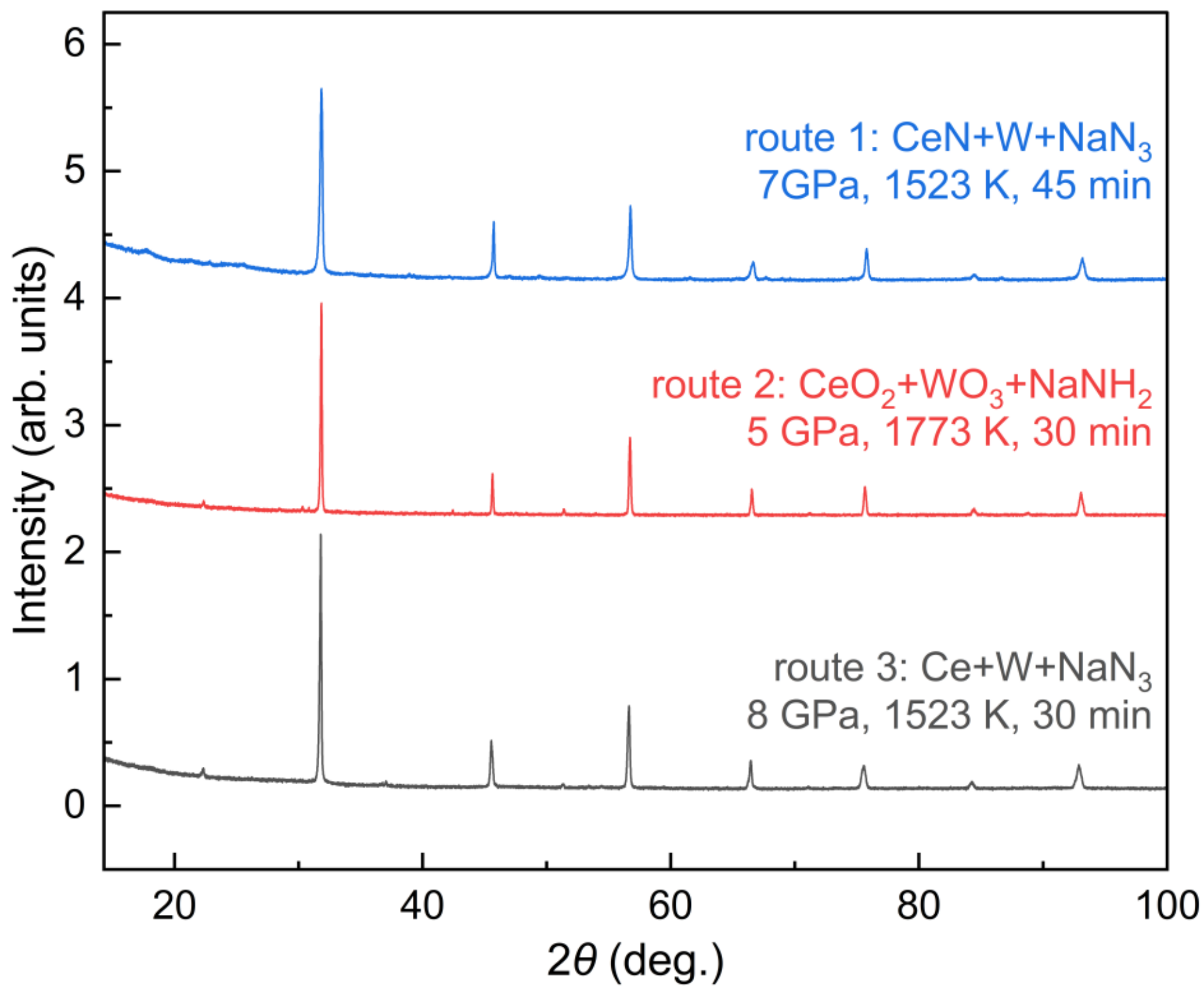

Fig. S15. Comparison of representative powder XRD patterns of $CeWN_3$ samples obtained using different synthetic routes. All three routes can yield $CeWN_3$ as the dominant phase, whereas the oxide/amide route using CeO_2, WO_3, and $NaNH_2$ achieves the target phase at a lower pressure of 5 GPa and provides improved experimental reproducibility and larger product yield. Therefore, this optimized route was adopted for preparing the samples used in the structural and physical property measurements. The patterns are vertically offset for clarity.

Table S1. Crystallographic parameters of $CeWN_3$ refined from synchrotron X-ray diffraction at RT.

Atom	WP	*x*	*y*	*z*	U_{iso} ($\times 10^{-2}$ Å^2)
Ce	4a	0.0148(2)	-0.0007(3)	0.2514(10)	0.38(1)
W	4a	0.4975(6)	-0.0001(7)	-0.0023(7)	0.39(1)
N1	4a	0.233(2)	0.225(2)	0.541(2)	0.70(3)
N2	4a	0.503(1)	0.048(1)	0.241(2)	1.10(3)
N3	4a	0.269(3)	0.750(3)	0.531(1)	3.23(6)

Space group $Pna2_1$ (Orthorhombic; No. 33); lattice constants a = 5.61525(1) Å, b = 5.62785(1) Å, c = 7.94437(2) Å. WP denotes the Wyckoff position. The site occupancy factors were fixed at unity. U_{iso} represents isotropic thermal parameters. Refinement reliability factors: R_{wp} = 6.80%, R_p = 4.78%, and χ^2 = 1.88.

Table S2. Refined Ce–N and W–N bond lengths and angles for $CeWN_3$ at RT.

Bond	Length (Å)	Bond	Length (Å)
W–N1	2.01(2)	Ce–N1	2.51(2)
	2.04(2)		2.68(2)
W–N2	1.95(1)		2.90(2)
	2.06(1)		3.19(2)
W–N3	1.94(3)	Ce–N2	3.077(8)
	2.07(3)		2.888(6)
			2.555(8)
**Bond**	**Angle (°)**		2.756(6)
W–N1–W	155.5(3)	Ce–N3	2.56(4)
W–N2–W	164.7(2)		2.99(4)
W–N3–W	172.8(6)		2.97(4)
			2.76(4)
